# Nanocomposite Polymer Electrolytes for Zinc and Magnesium Batteries: From Synthetic to Biopolymers

**DOI:** 10.3390/polym13244284

**Published:** 2021-12-07

**Authors:** María Fernanda Bósquez-Cáceres, Sandra Hidalgo-Bonilla, Vivian Morera Córdova, Rose M. Michell, Juan P. Tafur

**Affiliations:** School of Chemical Sciences & Engineering, Yachay Tech University, Urcuquí 100119, Ecuador; maria.bosquez@yachaytech.edu.ec (M.F.B.-C.); sahidalgo@yachaytech.edu.ec (S.H.-B.); vmorera@yachaytech.edu.ec (V.M.C.); rmichell@yachaytech.edu.ec (R.M.M.)

**Keywords:** polymer electrolytes, composites, biopolymers, zinc batteries, magnesium batteries, properties

## Abstract

The diversification of current forms of energy storage and the reduction of fossil fuel consumption are issues of high importance for reducing environmental pollution. Zinc and magnesium are multivalent ions suitable for the development of environmentally friendly rechargeable batteries. Nanocomposite polymer electrolytes (NCPEs) are currently being researched as part of electrochemical devices because of the advantages of dispersed fillers. This article aims to review and compile the trends of different types of the latest NCPEs. It briefly summarizes the desirable properties the electrolytes should possess to be considered for later uses. The first section is devoted to NCPEs composed of poly(vinylidene Fluoride-co-Hexafluoropropylene). The second section centers its attention on discussing the electrolytes composed of poly(ethylene oxide). The third section reviews the studies of NCPEs based on different synthetic polymers. The fourth section discusses the results of electrolytes based on biopolymers. The addition of nanofillers improves both the mechanical performance and the ionic conductivity; key points to be explored in the production of batteries. These results set an essential path for upcoming studies in the field. These attempts need to be further developed to get practical applications for industry in large-scale polymer-based electrolyte batteries.

## 1. Introduction

The development of new ways of obtaining energy from environmentally friendly materials is directly related to the need for developing devices capable of storing the power generated. Some devices are designed to store energy, such as rechargeable batteries, capacitors, sensors, and dye-sensitized solar cells (DSSC) [1]. Among these devices, batteries are the most used in everyday life around the world. The main disadvantage of the batteries currently in use, for example, lithium-ion batteries (LIBs), is that they can undergo thermal runaway, form protrusions, show low energy density, and low cycling efficiency [2,3,4]. Moreover, they are highly reactive, expensive, unsafe, and are a pollutant for the environment [5,6,7,8,9,10].

Some of the devices that could resolve the disadvantages identified for LIBs are redox flow batteries [11,12,13] and fuel cells [14,15,16], as they have no thermal runaway problem, and they are safe and less expensive. Among these energy storage options, zinc and magnesium are currently the multivalent ions in the sight of replacing lithium as the most reliable options to develop eco-friendly rechargeable batteries. Magnesium is the 8th most abundant metal in the Earth’s crust [17], while zinc is the 24th most abundant [18], with an estimated 2800 million metric tons (Mt) of zinc contained in the Earth’s crust [19]. Furthermore, magnesium and zinc can be recycled cheaply; in addition, they do not lose their physical properties [13,14] that contribute significantly to sustainability, aiming to reduce concentrate demand, energy consumption, and minimize waste disposal and pollutant emissions. On the other hand, it is known that lithium reserves present an amount of only 17 million Mt [20], and recycling lithium, which at present is heavily dependent on cobalt content, requires improvement due to environmental and economic concerns, besides the lower value of the recovered materials [21]. 

Zinc batteries present key features for battery’ performance including high volume capacity [22] and little redox potential [23]. On the other hand, magnesium batteries possess a low electrode potential and a high volumetric capacity, almost double the Li-metal value [24]. Besides, zinc and magnesium have lower reactivity, lower cost, low toxicity, and intrinsic safety [25,26,27,28,29], critical characteristics for developing sustainable energy storage devices. 

These batteries have a wide range of application fields in energy storage/release systems ranging from technological and military applications, to vehicles and wearable electronics [30,31,32]. To develop adequate energy storage devices for the end-users, one of the crucial features is whether the battery is only suitable for base station energy storage, or if it could also be employed for flexible devices. Hence, another point for the development of eco-friendly batteries is the physical state of the electrolyte. Currently, batteries use the electrolyte in a liquid state, which has safety, toxicity, flammability, and leakage drawbacks. In addition, other characteristics of current batteries, such as bulky design, electrode corrosion occurring at the interfaces, and dendrite growth on the metal electrode, reduce the capacity and life cycle of the device [33,34,35]. They can even lead to preferential nucleation and uneven currents during charging [36] and cause fires [37]. These issues are why current battery development strategies focus on using solid or gel-based electrolytes, improving the electrochemical properties. 

Polymer electrolytes (PEs) have the most far-reaching advantages among all types of solid-state and gel-based electrolytes. They stand out for their high flexibility and good performance [38,39,40]. However, the main problem of these electrolytes is that they present low battery efficiency, insufficient ionic conductivities for practical applications, insufficient electrochemical stabilities, poor mechanical strength, and substantial interfacial resistance [10]. Therefore, recent research has focused on incorporating inorganic phases; these hybrid systems have higher ionic conductivity and mechanical stiffness and are non-flammable [29,41,42].

From this approach, nanocomposite polymer electrolytes (NCPEs) were born. The first report mentioning the addition of inorganic fillers in PEs was reported by Weston et al. in 1982 [43]. These authors showed how the combination of both phases reduced the drawbacks of electrolytes that did not combine inorganic/organic phases. Since then, several papers have been published to assemble PEs and NCPEs for Zn and Mg that could be considered for industrial applications [2,10,44,45,46,47,48]. 

Biopolymers are in the main scope of this review, focusing on developing electrolytes that can be considered environmentally friendly and biocompatible. However, it is worth mentioning that for a biopolymer to be considered environmentally friendly, the resource and the production method are vital characteristics to be taken into account [49]. On the other hand, biopolymers are characterized by their natural abundance, cost-effectiveness, high solvent compatibility, and film-forming ability. Some reviews focusing on biopolymer electrolytes have been published [50,51,52]. However, nanofillers are a new approach discussed here to get a precedent for further research and obtain better results for practical applications among this type of polymers.

NCPEs must meet specific requirements to be suitable rechargeable batteries. The polymer that acts as the host should possess an amorphous or low crystalline nature [53]. The cation–polymer interaction must be sufficiently strong to promote dissolution but not so strong as to inhibit ion exchange [54]. The designed electrolyte should be able to take up polar groups with a high molecular weight in its chain apart from sufficient electron-pair donors for coordination with cations [53], to achieve a good performance in cationic transport number, more significant than with anionic to reduce the concentration gradients for obtaining repeated charge-discharge steps and high-power density [55]. 

**Figure 1:**
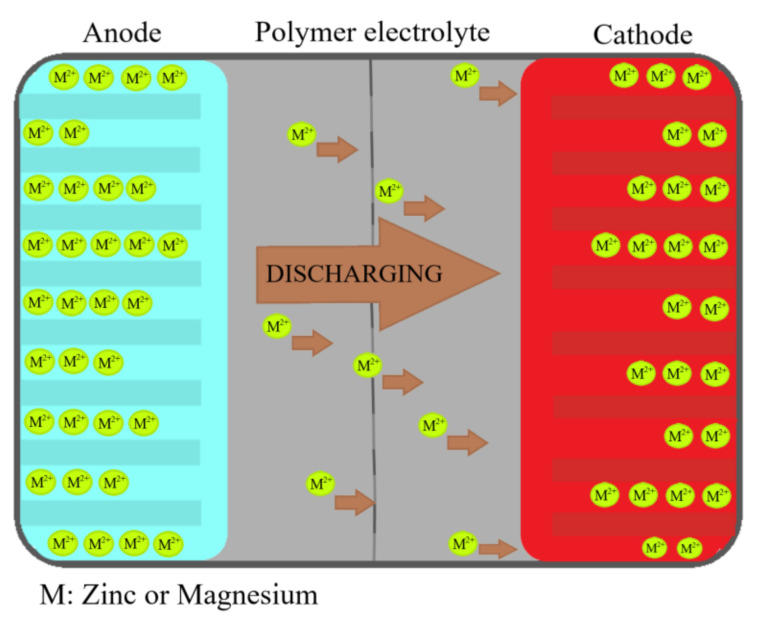
Schematic diagram of Zn-ion and Mg-ion battery discharge. Reproduced with permission from Renew. Sustain. Energy Rev., 65, Singh et al., Perspectives for solid biopolymer electrolytes in dye sensitized solar cell and battery application, 1098–1117, 2016 [50].

The electrolyte should undergo no net chemical changes of the battery. All Faradaic processes are expected to occur within the electrodes [56] since the electrolyte needs to be an inert battery part. Figure 1 presents the discharge scheme for a typical battery, whose circuit is closed so that electrons can get to the cathode. The performance level requires ionic conductivities values of at least 10^−3^ S·cm^−1^. Moreover, it needs to show the lowest glass transition temperature (T_g_) [54] possible, key for obtaining the highest conductivities, resulting in increased local segmental motion and, therefore, high diffusivity of the ions. 

For good performance, it is also relevant to fulfill some electrochemical properties [57], such as high decomposition potential, low interfacial resistance, as well as some degree of stiffness, high chemical and thermal stability, to be durable for a long time under the conditions in which the device in which it is to be used operates [53]. Finally, rentability in the production process is indispensable since the main goal of the development is to take it to the industrial scale. As reviewed so far, these are the most important characteristics to consider when studying electrolytes used in zinc and magnesium batteries.

In this review, the recent advances of NCPEs for magnesium and zinc rechargeable batteries are overviewed, with a particular interest in the results regarding their ionic conductivities, electrochemical stabilities, and general performances in battery systems. This field continues thriving; still, new aspects of the nanoparticles’ effects on the physical-chemical properties of the polymer electrolytes and their based power sources are ever discovered and need to be discussed to set an outline on future directions and challenges that come with the development of NCPEs for new batteries on worldwide demand.

## 2. Poly(vinylidene fluoride *co*-hexafluoropropylene)’s-Based Nanocomposite Polymer Electrolytes

Copolymerization is one of the most effective methods to improve the mechanical stability and electrical conductivity of materials [58,59,60]. Poly(vinylidene Fluoride-co-Hexafluoropropylene) polymer matrix (PVDF-co-HFP) has been extensively used for different purposes. It has an excellent performance in fuel cells, dye-synthetized solar cells, membrane distillation, and other electrochromic applications [61,62,63,64]. The block copolymer structure includes a crystallizable comonomer (-CH_2_-CF_2_-) and an amorphous HFP unit with a high dielectric constant (ε = 8.4), thanks to the presence of highly electronegative fluorine and the spontaneous alignment of C–F dipoles in the crystalline phases [65,66,67].

The copolymer presents a high solubility and lower crystallinity and glass transition temperature [68] than Poly(vinylidene Fluoride) (PVDF), making it a promising matrix for preparing nanocomposites, despite its non-biodegradability. Furthermore, the degree of crystallinity remaining in the system helps retain sufficient mechanical stability and structural rigidity to act as a separator between the battery’s electrodes [69]. At the same time, the amorphous phase can serve as the conductive medium.

### 2.1. Magnesium-Ion Conduction

Within the field of rechargeable batteries, several studies have applied PVDF-co-HFP as a component of the electrolyte. The most recent ones were compiled in a review article, which focuses on lithium-sulfur batteries [70]. However, as reported, the balance between industrial development and environmental protection makes it essential to develop high energy density and non-pollutant rechargeable batteries, with magnesium ion and organic electrode batteries as the directions for post-lithium batteries [71]. For reference, Table 1 lists some properties of the nanocomposites employed along with PDVF-co-HFP as electrolytes for magnesium batteries.

Maheshwaran et al. [72] studied the role that the salt added to the polymer had and how its amount affected the results. They developed a magnesium ion conducting gel polymer electrolytes (GPE) based on PVdF-co-HFP, magnesium triflate Mg(Tf)_2_, in ethylene carbonate (EC), and diethyl carbonate (DEC). The analysis of this polymeric electrolyte by X-ray diffraction (XRD) showed a decrease in the crystallinity with the addition of salt. Moreover, FT-IR confirmed that magnesium triflate could suppress the nonpolar α crystalline phase of PVDF. Consequently, the electrolyte offered a predominant ionic character with a total ion transport number close to unity, making it considered to a certain extent for batteries because it was freestanding and stable. However, its considerably low ionic conductivity made it inappropriate, although it became a precedent for what can be achieved.

Solid polymer electrolytes (SPE) with PVDF-co-HFP as a polymer matrix were also studied. Ponmani et al. [73] blended this matrix with Poly(vinyl acetate) (PVAc) and added magnesium perchlorate Mg(ClO_4_)_2_ salt. The SPE film was found to be flexible, and the maximum ionic conductivity found was 0.293 × 10^−3^ S·cm^−1^, obtained at 363 K. Cyclic voltammetry (CV) studies confirmed the Mg ion reversibility that demonstrated its conduction in the SPE. 

One of the first electrolytes that employed PVDF-co-HFP was a magnesium-ion conducting GPE, composed of 15% of PVdF-co-HFP, 73% of Mg(ClO_4_)_2_ in EC/propylene carbonate (EC/PC), and 12% silicon dioxide (SiO_2_) [74]_._ The cell in which this electrolyte was tested employed magnesium as anode and vanadium oxide (V_2_O_5_) as the cathode_._ The tests demonstrated low initial discharge capacity and poor cycling performances. These disadvantages could be attributed to high interfacial resistance at Mg anode [74]. The main problem identified from this research was the blocking of the charge transfer reaction, highlighting that further research on the interface should be conducted so that the cycling performance could be improved to a practical level.

Magnesium oxide (MgO) showed beneficial features in inducing consistent improvements in liquid electrolyte retention and the overall chemical, physical, and electrochemical properties in the work performed by Pandey et al. [79]. They presented novel research dispersing PVdF-co-HFP with nanosized MgO particles. It was analyzed by XRD patterns, obtaining a semi-crystalline structure with predominant peaks in 2θ = 14.6, 17, 20, 26.6, and 38°. These changes in the peaks showed the reduction in crystallinity of the PEs, caused by the entrapment of liquid electrolytes. FT-IR spectroscopic analysis was conducted to look over the ion-polymer interaction and the conformational changes, confirming the reduction of crystallinity. The T_g_ was observed at −65 °C for pure PVDF-co-HFP film, while with the addition of magnesium oxide, the value came down to −90 °C. 

Electrodes play an important role when performing efficiency analysis. Pandey et al. [75] demonstrated the previous NCPE in a prototype cell of magnesium and multiwalled carbon nanotubes (MWCNT) composite as the negative electrode and the corresponding positive one with vanadium pentoxide (V_2_O_5_). The rechargeability of the cell was enhanced by substituting magnesium with Mg–MWCNT composite as the negative electrode. The discharge capacity faded away after ten cycles, attributed to the passivation of the negative electrode. Nevertheless, the electrolyte showed to be free-standing and flexible, with enough mechanical strength.

**Figure 2:**
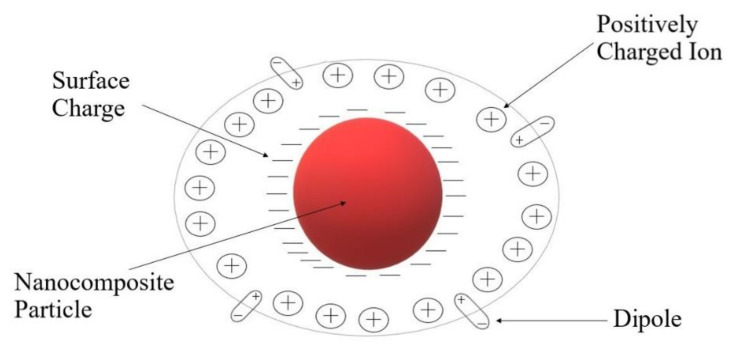
Schematic representation of the space charge and local electric field formation around a nanocomposite particle.

The role of fillers has been shown to be of great importance. These nanocomposites can form space-charge regions and induce a local electric field. This phenomenon was first approached by Kumar [86], who revealed the electric charge and area associated with the particle interact with the structure of the liquid electrolyte, provoking the space-charge region. It can be described as containing free electrons at the surface of the nanocomposite, and cations along with dipoles at the adjacent double-layer balance the surface electronic charge (Figure 2). Magnesium oxide is known to be slightly electronegative in nature. In the systems studied, a reversible reaction between the magnesium oxide and the magnesium (II) ion took place and formed the space-charge region, giving place to the MgO:Mg^2+^ species [75].

**Figure 3:**
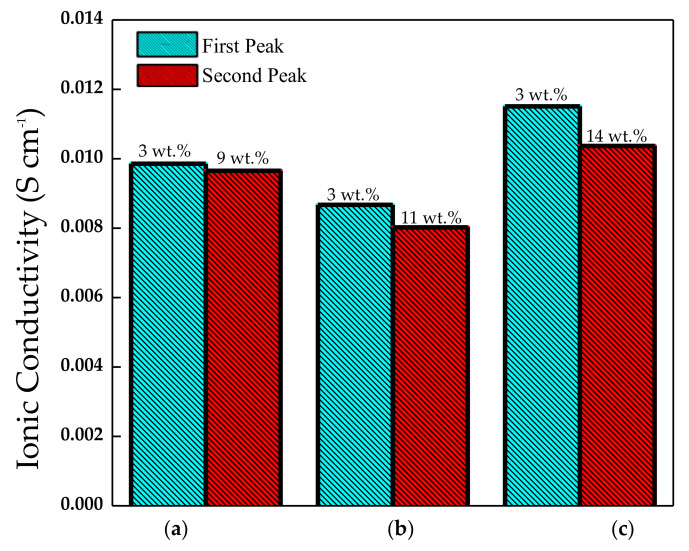
Room temperature conductivity peaks of composite gel polymer electrolyte films vs filler content: (**a**) nano-sized MgO, (**b**) micro-sized MgO, and (**c**) nano-sized SiO_2_. Prepared from data in [81].

Magnesium oxide nanoparticles were combined with nano-sized silicon dioxide in a novel electrolyte by Pandey et al. [81]. When relating conductivity to filler content (Figure 3), the presence of two conductivity maxima was noticed, explained by the dissociation of ion aggregates/undissociated salt into free ions with the addition of filler particles (the first peak). The second maximum was described using the composite effect and based on a conducting interfacial space-charge double layer between the filler particles and the GPE. This local field was responsible for enhanced Mg^2+^ ion motion and enhanced transport number up to the addition of ∼10 wt % of MgO. The cationic transport number measurements (t_+_) also showed essential results, in which the best improvement was obtained by the presence of MgO particles (∼0.44). On the other hand, with the addition of SiO_2_ dispersion, the t_+_ value did not increase substantially. Finally, for this polymer, it was pointed out that the nano-sized MgO supported the cationic motion. In contrast, the nano-sized SiO_2_ supports the anion conduction in the filler/gel electrolyte interfacial regions.

Nanosized silicon dioxide was ultimately tested with the addition of molybdenum trioxide (MoO_3_) as the positive electrode in a posterior study [76]. This cell showed a discharge capacity of ∼175 mAh·g^−1^ for an initial ten charge-discharge cycles. In addition, it presented the same conductivity value as the last cell. Finally, good thermal stability with a single-phase behavior was presented at a temperature range from −70 °C to 80 °C. Enhanced conductivity was attributed once again to the space-charge layers formed between the filler and GPE. 

**Figure 4:**
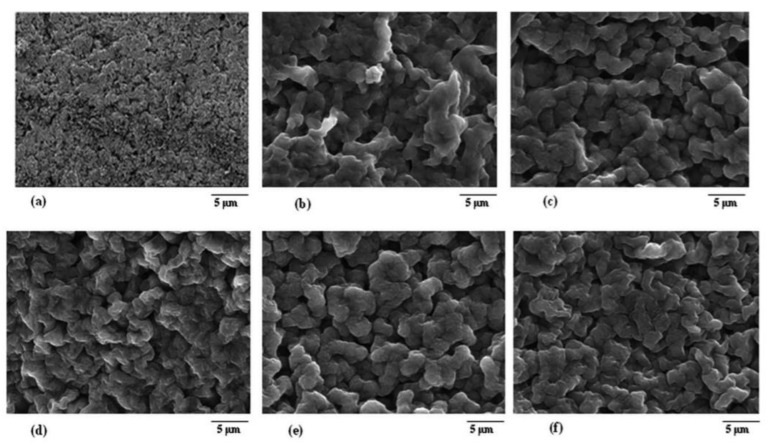
FESEM images of pure (**a**) PVdF-co-HFP film and nanocomposite GPE films containing (**b**) 0 wt %, (**c**) 6 wt % Al_2_O_3_, (**d**) 30 wt % Al_2_O_3_, (**e**) 6 wt % MgAl_2_O_4_, and (**f**) 20 wt % MgAl_2_O_4_. Reproduced with permission from Polym. Compos., 40, Sharma et al., Magnesium ion-conducting gel polymer electrolyte nanocomposites: Effect of active and passive nanofillers, 1295–1306, 2019 [77].

The effect of active and passive nanofillers, along with the copolymer, was studied for Mg NCPEs by Sharma et al. [77], incorporating Mg-triflate salt mixed with EC and PC, entrapped in PVDF-co-HFP. Aluminium oxide (Al_2_O_3_) and Mg aluminate (MgAl_2_O_4_) were used as passive and active fillers. The reduction of crystallinity was achieved as expected and confirmed by XRD, Field emission scanning electron microscopy (FESEM), and Differential Scanning Calorimetry (DSC) studies. By FESEM (Figure 4), it was observed that undispersed GPE showed larger grain sizes and possessed uniformly distributed pores. The incorporation of nanofillers in the undispersed GPE changes its morphology substantially. It was further proved that as the number of fillers increases its porosity leads to the entrapment of liquid electrolyte in the pores (demonstrated by the fillers not being seen in the NCPE (Figure 4c,f)), which further enhanced the ionic conductivity of the NCPE. The addition of the passive filler conferred the cell to have a relatively good mechanical stability and thermally stability up to 100 °C. The active filler ensured an improvement in the ion transport number. The obtained electrochemical stability window (ESW) was key, showing their potential as electrolytes in ionic devices.

The latest report of PVDF-co-HFP electrolyte for magnesium batteries known so far implemented zinc oxide (ZnO) as nanofiller along with Magnesium chloride (MgCl_2_) as ionic salt [82]. A transport number of 0.99 was achieved; the current change indicated that conductivity in the NCPE was predominantly ionic. PVDF was incorporated without copolymerizing it with HFP in a magnesium NCPE, along with MgO as a nanofiller [80]. The optimum nanofiller concentration (3wt %) was chosen to be the most suitable one with a conductivity of 1.04 × 10^−4^ S·cm^−1^. On further increase in nanofiller concentrations, the ionic conductivity value decreased. Thermal stability and reduced melting point temperature were confirmed through thermogravimetric analysis (TGA) and XRD. Magnesium oxide nanoparticles enhanced the ionic conductivity and dielectric constant, confirmed by complex impedance spectroscopy [80]. The authors recently presented similar results with the addition of zinc oxide particles [83].

The results presented until now (Table 1) confirm the enhanced high ionic conductivity present in PVDF-co-HFP nanocomposite polymer electrolytes compared to the SPE systems and better thermal and mechanical stability compared to liquid systems. The enhancement in conductivity may be caused by the presence of the nanoparticles, facilitating the new kinetic path for ionic transport and polymer segmental motion. However, another conclusive characteristic is that when a specific percentage of nanofiller is added, a decrease in ionic conductivity is observed. Excessive fillers could provoke this in the NCPE that may trigger the formation of ion pairs and ion aggregation, such as the non-conducting phase presented as an electrically inert component blocking ion transport. So far, they are probably one of the best options to study and meet all the requirements for future use instead of lithium-ion conductive systems.

### 2.2. Zinc-Ion Conduction

Besides magnesium, zinc presents many advantages associated with zinc chemicals, as batteries of high specific/volumetric energy density can be fabricated. Ionic radii of Zn^2+^ (74 pm) and that of Li^+^ (68 pm) are quite comparable, but Zn^2+^ has twice as much charge as Li^+^ cation [87]. Furthermore, the natural resources of zinc are plentiful, and its stability makes it able to be handled safely in oxygen and humid atmosphere. The so-mentioned dielectric constant of PVDF-co-HFP is also known to generally assist in more significant ionization of zinc salts and then provide a high concentration of charge carriers. Consequently, Table 2 lists some properties of the nanocomposites employed along with PDVF-co-HFP as electrolyte for zinc batteries. 

Tafur et al. [88] studied GPEs composed of PVdF-co-HFP with different ionic liquids, with and without zinc triflate salt. From attenuated total reflectance-Fourier transform infrared (ATR-FT-IR) and XRD spectroscopies, it was deduced that incorporating the ionic liquid and salt to the matrix produced more amorphous and polar membranes when comparing it to the original PVDF-co-HFP film. Besides, the electrical properties had shown to be dependent on the ionic liquid employed, aspect confirmed by measurements on ionic conductivity, impedance, and voltammetry. This report also studied the influence of the ionic liquid type on the performance of the GPE for Zn batteries. From ionic conductivity, impedance, and voltammetry measurements, changes in the results were observed when the salt was not added, or the added quantity was too low, indicating that the salt is the important charge carrier independent of the ionic liquid.

In another report, Tafur et al. [95] designed a battery employing manganese dioxide (MnO_2_) as the cathode, zinc as the anode, and the GPE was assembled by the use of PVDF-co-HFP including 1-Butyl-3-methylimidazolium trifluoromethanesulfonate, 99% (BMIM Tf), and zinc triflate (Zn(Tf)_2_). The electrolyte was then analyzed by X-ray photoelectron spectroscopy and Energy Dispersive X-Ray techniques. The remarkable results from the study showed the charge storage mechanism, which began with the reduction of Mn^4+^ to Mn^2+^ species, at the same time as Zn^2+^ cations, together with triflate ions, intercalate the cathode material during the discharge process. In the recharge process, it was evidenced that Mn^2+^ species returned to the positive electrode, and they were oxidized mainly to Mn^4+^. Moreover, Mn^2+^ was not reduced to Mn^0^ in the anode during the recharge process. Nevertheless, in every completed process, Zn^2+^ cations were not expulsed, remaining inside the electrode, probably stabilized by the triflate anions, which were not expulsed either. Besides, with the addition of an ionic liquid to the GPE, it was observed that the interaction between the zinc-ion and PVDF chains became weaker, enhancing ion mobility. 

A zinc battery was designed with an SPE [89] based on PVDF-co-HFP with zinc triflate_,_ obtaining a low crystallinity elucidated by XRD and scanning electron microscopy (SEM). CV of the SPE curve was flat without the presence of peaks. This fact indicated that the film presented excellent stability. No decomposition occurred in the operating voltage range and ESW of 3.45 V. These results were improved in a recent study performed by Liu et al. [90], where 1-ethyl-3-methylimidazolium trifluoromethanesulfonate (EMITf) was incorporated along with the PVDF-co-HFP membranes and zinc triflate salt. The ionic liquid has shown to reduce the crystallinity, enrich the nanopores’ structure, and enhance the electrical and electrochemical properties of the electrolyte membranes. The ionic conductivity was enhanced by one order of magnitude. The electrolyte membrane was able to sustain a high thermal decomposition temperature of ~305 °C, and thus its mechanical performance was sufficient for considering it for practical applications.

Nanocomposites became first used with PVDF-co-HFP by Muda et al. [94], who designed a cell composed of Zn + ZnSO_4_·7H_2_O + polytetrafluoroethylene (PTFE) as the anode, MnO_2_ + PTFE as the cathode, and the electrolyte composed by PVDF-co-HFP as host polymer, ammonium trifluoromethane sulfonate (NH_4_CF_3_SO_3_) as ionic salt with silicon dioxide as filler. Herein was observed the so mentioned existence of two conductivity maxima at the concentration of 1 and 4 wt % of SiO_2_, in this case, attributed to two percolation thresholds in the NCPE. The voltage was able to achieve a value of ~1.50 V to ~1.29 V. The assembled cell performed fairly well when discharged at the low current drain or with high load resistance, showing suitability for low current drain applications.

**Figure 5:**
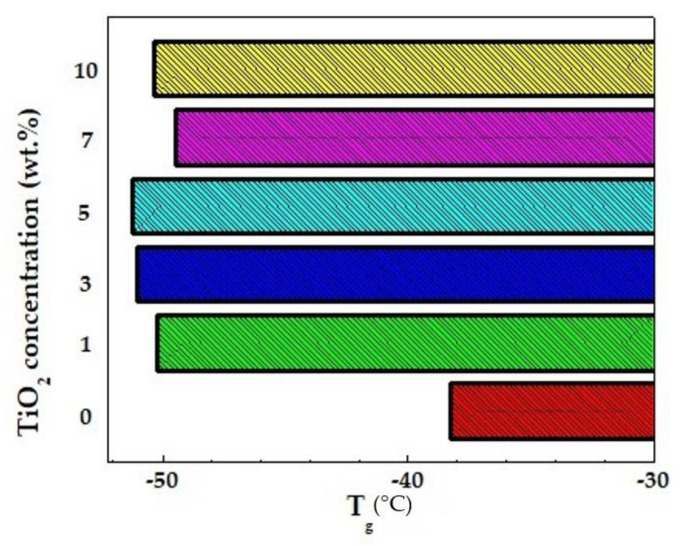
T_g_ (°C) of the NCPE systems vs the TiO_2_ content. Prepared from data in [91].

Titanium dioxide was incorporated in an NCPE designed by Johnsi et al. [91]. Differential scanning calorimetric results confirmed that with the addition of TiO_2,_ a reduction in the degree of crystallinity and the T_g_ value was obtained. The glass transition temperature is a fundamental parameter to grasp the structural changes of PEs occurring under various thermal conditions. The effect of filler content on the position of glass temperature could be evaluated from Figure 5. When more TiO_2_ was added, these values increased slightly instead, which could be caused by the possible agglomeration of an excess amount of nanofillers. Furthermore, there was also evidence to ascertain that the decrease of the T_g_ value reflected an increase in flexibility of polymer chains, provoking enabling fast ion conduction within the NCPE system. The low value of conductivity limited the applications that could be obtained for this electrolyte.

**Figure 6:**
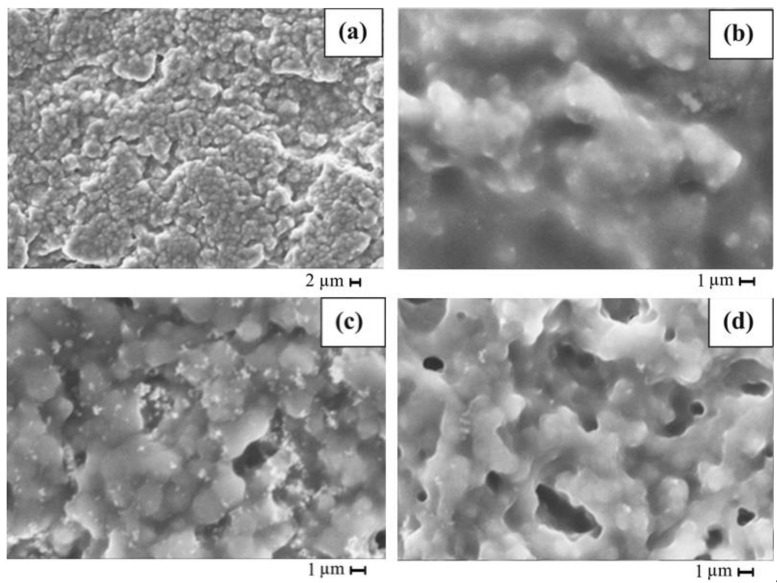
SEM micrographs of EC–PC–Zn(Tf)_2_+PVdF-co-HFP (**a**) gel polymer electrolyte (magnification, ×1000); (**b**) gel electrolyte with magnification ×5000; and (**c**) its nanocomposites dispersed with ZnO particles of 10 wt % (magnification, ×5000); and (**d**) 25 wt % (magnification, ×5000) Reproduced with permission from J. Solid State Electrochem., 16, Sellam, Enhanced zinc ion transport in gel polymer electrolyte: Effect of nano-sized ZnO dispersion, 3105–3114, 2012 [93].

Active fillers were studied by Hashmi [93] with an electrolyte composed by the addition of 1.0 M solution of zinc triflate in EC/PC immobilized in the host polymer and ZnO nanofiller. The morphological/structural changes for this gel electrolyte were monitored using SEM and XRD techniques. The micrographs obtained (Figure 6) showed that the texture and morphology of the gel polymer system had also been modified, revealing smaller crystallites and pores. For the highest amount (∼25 wt %) sample, ZnO particles appeared to be disappeared in the SEM picture (Figure 6b), probably due to the polymer fully covering the particles, changing the texture of polymer network. On the other hand, with 10 wt % of filler, white spots could be seen, indicating the presence of nanoparticles in the gel network, forming a separate phase. Dark regions showed the micron-sized porosity from the micrographs corresponding to the undispersed GPE, where the liquid electrolyte could be retained. Thus, the polymer films established a semi-crystalline nature with enough porosity to maintain the ionic liquid in the electrolyte. For this gel, the increase in ionic conductivity with the dispersion of ZnO did not signify more than an order of magnitude, obtaining that for the undispersed sample, its value was ∼6.7 × 10^−3^ S·cm^−1^. Besides, with the increasing of temperature, for the sample of ∼10 wt % ZnO particles, it offered ionic conductivities of 3.7 × 10^−3^ S·cm^−1^ at 30 °C and 1.4 × 10^−2^ S·cm^−1^ at 85 °C, which gave a precedent for potential application as an electrolyte in zinc batteries and other electrochemical applications over a wider temperature range. Furthermore, for this NCPE, a local electric field was detected due to the reversible reaction between ZnO and Zn^2+^, homologous to the one previously presented in Figure 1.

Johnsi et al. [57] continued their work with passive fillers. They constructed a flexible, free-standing, transparent film composed of [75 wt % PVDF-co-HFP:25 wt % ZnTf_2_]-x wt % cerium dioxide (CeO_2_) where x = 1, 3, 5, 7, and 10, respectively. The film’s detailed FT-IR spectral analysis indicated the feasibility of complexation between the host polymer matrix and the salt and nanofiller. The decomposition voltage that reached a range of 2.4 to 2.7 V was a key result for this cell. Johnsi’s et al. [92] work continued with implementing zirconium dioxide (ZrO_2_), with the same proportion of PVDF-co-HFP and ZnTf_2_, with 7 wt % nanofiller. The obtained value of ionic conductivity represented an increase in an order of magnitude. XRD confirmed an amorphous phase present in the matrix. The cell achieved an ESW of 2.6 V with thermal stability up to 300 °C. The resulting cell exhibited many attractive and stable discharge characteristics for room temperature applications.

**Figure 7:**
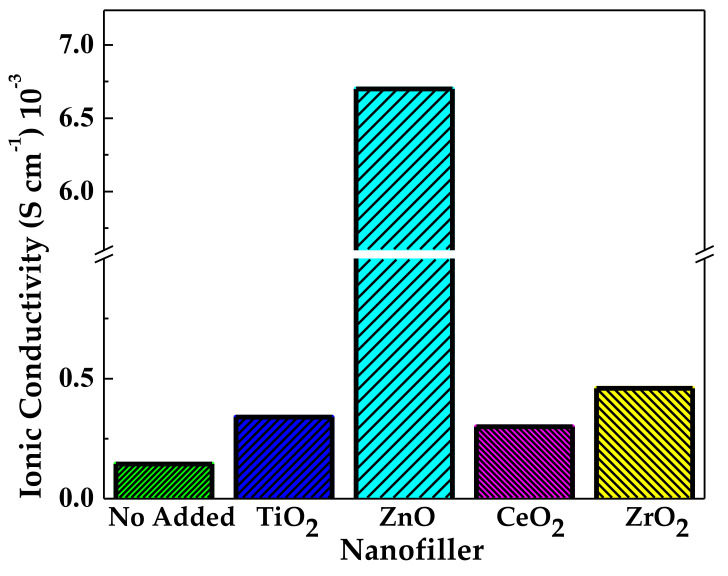
Plot of nanofiller versus ionic conductivity (×10^−3^) for nanocomposite polymer electrolyte based on PVDF-co-HFP and zinc triflate salt. Prepared from data in [57,90,91,92,93].

From the analysis presented in this section, it could be concluded that the addition of active fillers has shown more promising results for the applicability of NCPEs, than the ones with passive fillers. This assumption can be seen in Figure 7. The reports about PVDF-co-HFP, with the addition of zinc triflate salt and various nanofillers, are presented versus their ionic conductivity obtained. Whereas the first bar, representing the NCPE without nanofillers, has a shallow and almost imperceptible value, the bar corresponding to the ZnO nanofiller has a clear advantage over the other nanofillers considered passive. This assumption is explained by the fact that zinc oxide nanoparticles have been shown to participate in the ionic conduction process. Hence, they deserve special attention for further studies on the field, even with other polymers and ionic salts.

## 3. The Poly(ethylene Oxide)’s Based Nanocomposite Polymer Electrolytes

Poly(ethylene oxide) (PEO) has been the focus of attention for many researchers among various polyethers because it is considered to be the best solvent medium for various ionic salts [96], and it is known to possess relatively high electrochemical stability [97]. It is water-soluble and in a semi-crystalline state at room temperature [98]. It presents a single helical structure, which also supports ionic conduction [99], favoring fast ion transport for the electrochemical processes in the batteries. The main chain of the polymer, known to be polar and flexible, owns vital electron-donating ether-oxygen groups, dissociating the salt, and generating carrier ions. These ions can migrate through the amorphous region of the polymer employing interchain/intrachain segmental motion. However, its high degree of crystallinity makes it necessary to incorporate metal salts that impede crystallization [100]. PEO shows a low ionic conductivity of 3.32 × 10^−9^ S·cm^−1^ at pure state [101], a fact that could be enhanced by the addition of nanofillers, along with other approaches discussed in this section. A review article was recently published by Feng et al. [102], where the interaction of ceramic fillers on the performance of PEO in lithium batteries is deeply studied. The authors concluded that composite SPEs are one of the most efficient ways to improve the electrolytes’ ionic conductivity.

### 3.1. Magnesium-Ion Conduction

The primary purpose of the subsequent studies has been to elucidate the ion transfer mechanism in the nanocomposite polymer system and enhance the ion conductivity and mechanical strength. The present section discusses how the addition of nanocomposites decreases the degree of crystallinity in these electrolytes. Table 3 refers to the properties of the nanocomposites employed along with PEO as electrolytes for magnesium batteries. 

**Figure 8:**
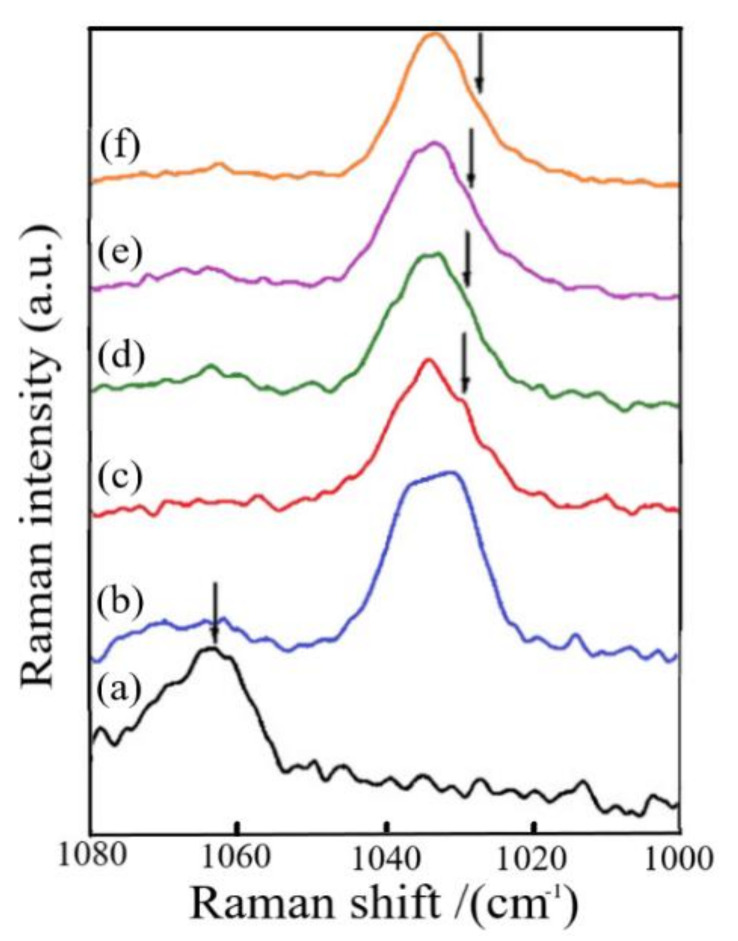
Raman spectra of (**a**) PEO pure film, (b) PEO25·Mg(Tf)_2_ complex, and PEO25·Mg(Tf)_2_ + x wt % EMITf system for (**c**) x = 5, (**d**) x = 10, (**e**) x = 20, and (**f**) x = 30 for spectral region of 1000–1080 cm^−1^. Reproduced with permission from Electrochim. Acta, 56, Kumar et al. Ionic liquid mediated magnesium ion conduction in poly(ethylene oxide) based polymer electrolyte, 3864–3873, 2011 [103].

The studies involving PEO for magnesium-ion conduction began complexing magnesium triflate and incorporating EMITf ionic liquid, reported by Kumar et al. [103]. The ionic liquid happened to be vital in mediating the Mg^2+^ ion conduction and the gradual enhancement in the Mg^2+^ ion transport number. Raman studies evidenced the interaction of imidazolium cations with ether oxygen of PEO (Figure 8). The peak at 2871 cm^−1^ was found to be affected due to the complexation of PEO with Mg(Tf)_2_ salt and the addition of ionic liquid. A peak appeared at 2848 cm^−1^ in curve b indicated the conformational changes of PEO chains after its complexation with Mg-salt. Besides, the PEO peak of 2871 cm^−1^ decreased and almost disappeared for the higher ionic liquid content. An additional peak (shoulder) appears at ∼1025 cm^−1^ (Figure 8c–f), presumably because of the free triflate anions from EMITf. Such anions would be free only when EMI+ ions have the possibility of interaction with ether oxygen of PEO. Consequently, it has been shown to play an essential role in substantially enhancing the cation transfer value. 

The approach of adding nanocomposites to PEO began with the report presented by Sundar et al. [106], who created an SPE of PEO with MgCl_2_ as electrolytic salt and boron oxide (B_2_O_3_) as the filler. DSC and FT-IR characterized this cell. The best ionic conductivity was achieved with 2 wt % B_2_O_3_. The cell was assembled by adopting Mg as anode and MnO_2_ as cathode, sandwiching the SPE between the electrodes. It got an open circuit voltage (OCV) of 1.9 V. The low ionic conductivity obtained made this cell unsuitable for practical applications. It indicated that this type of matrix could be considered for its cell performance, using fillers in the nanoscale. 

In the work presented by Shao et al. [108] comprised a novel electrolyte based on PEO, magnesium borohydride (Mg(BH_4_)_2_)_,_ and MgO nanoparticles. A key feature presented by this work was a high coulombic efficiency of 98% for Mg plating/stripping and high cycling stability. The experiments and modeling performed established a correlation between improved solvation of the salt and solvent chain length, chelation, and oxygen denticity. A further development in experimentation with this polymer revealed that it could be used in NCPE for other multivalent chemistry to delineate the ionic association and solvation interactions within these electrolytes.

**Figure 9:**
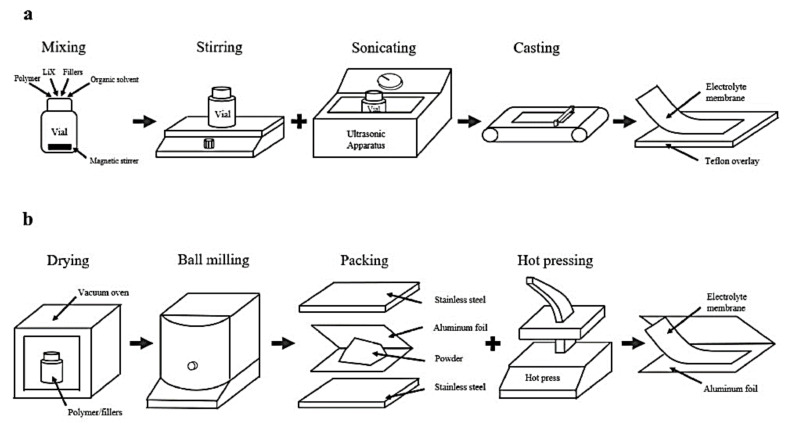
Schematic procedures for preparing solid polymer electrolytes by (**a**) solvent casting technique and (**b**) thermocompression. Reproduced with permission from Nano Converg., 8, Feng et al.PEO based polymer-ceramic hybrid solid electrolytes: a review, 2, 2021 [102].

Another approach for a casting technique by dry/solution free hot press is presented in the following studies as an alternate way of synthesis since no organic solvent as the medium for mixing ingredients is needed, which is the most significant difference from solvent casting [102]. The main steps for solvent-casting and hot-pressing are shown in Figure 9a,b. Thermocompression avoids contact with air during the process, which results in more stable productions. On the other hand, the solvent-casting method can disperse the ceramic fillers more uniformly, resulting in more ductile films. Besides, the residual liquid in this procedure can act as a plasticizer for the further performance of the SPE. These features need to be taken into consideration depending on the application the electrolytes will have.

Thermocompression was implemented in the work done by Agrawal et al. [104]. The assembled electrolyte, composed of phases, has an SPE film of PEO and Mg(ClO_4_)_2_ salt, the first phase host matrix with a conductivity of ∼2.77 × 10^−6^ S·cm^−1^. The second phase had MgO nano/micro-sized particles as active fillers and TiO_2_/SiO_2_ nano-sized particles as passive fillers. The dispersion increased the room temperature conductivity of the SPE host by ∼3–5-fold. The values of cation transport, however, remained in the range 0.21–0.30. 

**Figure 10:**
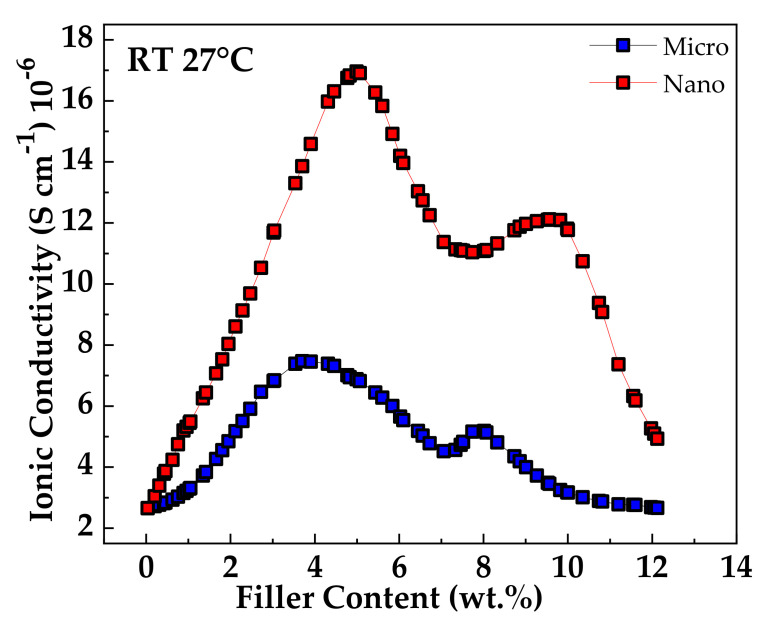
Active filler concentration-dependent conductivity variation for NCPE films: [80PEO: 20Mg(Tf)_2_] + xMgO micro/nano. Reproduced with permission from Mater. Chem. Phys., 139, Agrawal et al., Investigations on ion transport properties of hot-press cast magnesium ion conducting Nano-Composite Polymer Electrolyte (NCPE) films: Effect of filler particle dispersal on room temperature conductivity, 410–415, 2013 [101].

Casting by hot-press technique was performed as the synthesis process in the research developed by Agrawal et al. [101], with a primary phase host composed of PEO and magnesium triflate and micro/nano-sized materials TiO_2_ (passive filler) and MgO (active filler). The employment of all of these substances together achieved an enhancement in the room temperature conductivity of the SPE host. Characterization was performed with XRD, FT-IR, DSC analysis. The total ionic transference number data (∼0.98–0.99) showed the predominantly ionic character of the materials employed. Analyzing the concentration vs. log of conductivity (Figure 10) obtained two maximum peaks that suggested the presence of two conductivity mechanisms in the system, previously discussed in this report with reference [81]. The first peak could be related to dissociating undissociated salt and ion aggregates (if existed) into free ions. The second s-peak was then attributed to forming a high conducting interfacial space-charge double layer around insulating filler nano-particles that could correspond to a filler particle percolation threshold. It is also concluded that nanofillers were more effective in increasing the cation transport number than micro fillers, increasing the cation transport number t_+._

One of the latest reports on electrolytes based on PEO for Mg batteries was reported by Zaky et al. [105], with the incorporation of Mg salts treated with gamma irradiation to improve the PEO-Mg salt particle sizes. The electrical conductivity evaluated was more than three orders of magnitude than pure PEO, with a maximum value of 3.63 × 10^−3^ S·cm^−1^. The optimum ionic conductivity of MgO in the irradiated sample was obtained with 20 mL, while 30 mL was the best for un-irradiated. The addition of MgO also improved the electrochemical potential window to about −3.2 to 4.4 V. 

### 3.2. Zinc-Ion Conduction

In terms of zinc batteries, some works have been developed with the use of PEO. They are summarized in Table 4. For this metal, the first attempts were also designed without the addition of nanofillers. Therefore, an SPE with the addition of zinc chloride (ZnCl_2_) was developed by Carrilho et al. [109] in a cell composed of zinc and niobium pentoxide (Nb_2_O_5_) as electrodes. Their studies were performed at a temperature of 55 °C, obtaining a conductivity of 2.7 × 10^−4^ S·cm^−1^ and a cationic transference number value of 0.44 ± 0.05. 

The cell testing showed a decrease in the cell voltage without attaining any constant value. This result suggested the discharge product was the result of a topochemical insertion. The capacity retention of the cell was observed to be very poor. After the second cycle, the cell was not able to retain its charge. Galvanostatic/potentiostat cycles were performed at a lower time (in discharges) and fixed potentials (in charges) to improve the latter result. After this, shallower discharges were obtained, resulting in a longer cycle life, with a less marked decrease in cell capacity at a constant voltage. Lifetime evaluation for the studied cell was 4.9 years if maintained at 55 °C, under non-operating conditions.

Agrawal et al. [114] designed two cells that employed PEO and NH_4_HSO_4_ and SiO_2_ for the electrolyte that performed in two types of cells: MnO_2_ + C and PbO_2_ + V_2_O_5_ + C as cathodes, respectively. The researchers achieved an enhancement in the room temperature conductivity of polymer electrolyte approximately by an order of magnitude. Furthermore, it obtained a substantial increase in the mechanical strength of the films. The OCV was found to be in the range of 1.5–1.8 V for both batteries. The cell potential was stable through the discharges, but it discharged more quickly during higher current drain or low load resistance.

Gamma (γ) irradiation was presented as a novel technique to inhibit the crystalline phase in an electrolyte composed of PEO and ZnCl_2_ as salt, with the addition of nanosized TiO_2_ grains, by Turković et al. [112,113]. The polymer was subjected to γ-radiation from a Co-60 source. This approach was attempted since high-energy radiation could induce interchain linking of the polymer, inhibiting the crystalline phase in the polymer matrix. Small-angle X-ray scattering (SAXS) was recorded simultaneously with DSC, and wide-angle X-ray diffraction (WAXD) analyses were performed. Thanks to these techniques, it was obtained that the nanostructure of the γ-irradiated electrolyte changed during the crystalline-amorphous phase transition to a highly conductive superionic phase. Reduction in the T_g_ was observed, and ionic conductivity was enhanced, two desired changes in these processes. The conductivity of the nanocomposite prepared with irradiated powder ensured an improvement of two orders of magnitude compared to its homologous without irradiation.

An NCPE film was prepared using an SPE composed of PEO and zinc trifluoromethanesulfonate (Zn(Tf)_2_) and then incorporating Al_2_O_3_ nano-filler particles by Karan et al. [111] using a completely dry hot-press cast technique. The complexation of the salt and the dispersal of filler in the host substantially increased the amorphous region, which supported the increase in ionic conductivity and cationic transport number. Nevertheless, the obtained values need to be improved for possible applications in high-energy batteries. 

As another approach for employing nanocrystalline Al_2_O_3_, PEO was blended with polypropylene glycol (PPG) and Zn(Tf)_2_ as dopant salt by Nancy et al. [110]. This matrix resulted in an enhanced ionic conductivity of one magnitude, compared to the previous work where this nanofiller was employed with alone PEO. This feature caused segmental flexibility and an increase in the amorphous phase. The SEM, XRD, and DSC measurements showed that conductivity was controlled by segmental motions of the polymer chain and ion hopping mechanism at Lewis acid-base sites and at elevated temperatures exhibited Arrhenius behavior which was satisfactorily explained by free volume theory.

Zinc ferrite nanoparticles were presented as a relatively new approach for NCPEs by Agrawal et al. [116] This nanofiller has been widely used in technological applications because of its high magnetic permeability in the radio frequency region and low core loss. In their research, they posited the changing of the bonding behavior of the system when compared to the original PEO. Herein, the decrease in crystallinity was confirmed by the DSC study of the system. The presumed hopping mechanism between coordinated sites, local structural relaxation, and segmental motion of the polymer was stated because of the increase of ionic conductivity with temperature. The rise in ~3–4 order of magnitude of ionic conductivity concerning the pure polymeric host confirmed its promising results for electrolyte applications.

The hot-press technique was attempted with ZnO active fillers by Karan et al. [115], who composed a two-layer electrolyte. The first layer obtained the highest conductivity of 1.09 × 10^−6^ S·cm^−1^. A 5 wt % of ZnO revealed an optimum conduction composition with a conductivity of ~1.84 × 10^−5^ S·cm^−1^, meaning that the filler’s dispersal causes an enhancement of one order. The overall enhancement of four orders of magnitude from the pure PEO was obtained. The battery in which the films were assembled performed well under a low current drain state. 

**Figure 11:**
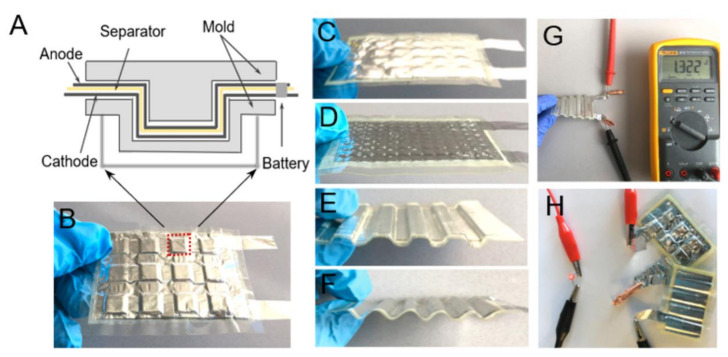
(**A**) Schematic of the mold used for plastic deformation studies. (**B**−**F**) Different plastically deformed shapes of Zn battery with solid-state biomimetic electrolyte. (**G**) The open-circuit voltage of Zn/PZB-931/γ-MnO_2_ battery with square wave shape plastic deformation. (**H**) LED light powered by the two serial structural batteries. Reproduced with permission from ACS Nano, 13, Wang et al., Biomimetic Solid-State Zn^2+^ Electrolyte for Corrugated Structural Batteries, 1107–1115, 2019 [117].

Ultimately, the outlook of incorporating branched aramid nanofibers (BANFs) to PEO was investigated by Wang et al. [117]. This combination enhanced the effective suppression of dendrites and fast cation transport due to the high stiffness of the BANF network combined with the high ionic conductivity of soft PEO, resulting in high tensile strength. The resulting battery showed the ability to withstand elastic deformation during bending and plastic deformation and remain functional (Figure 11). There have been different types of batteries that have shown to be capable of elastic deformations [118,119,120]; the ability to withstand plastic deformations while retaining the charge storage functions was a novel feature presented in this work. These features set it apart from other promising storage devices, improving the safety of the battery and its resistance to impact. 

The review in this section of the paper has shown that active fillers are supposed to be the first choice when choosing ceramic additives for PEO electrolytes. Moreover, the most optimized concentrations for these nanofillers are between 10–20 wt % to obtain the highest conductivity of each medium. Besides, the mechanical strength is shown to get better with the doping of ceramic particles. Moreover, the interfacial stability is assumed to be improved due to the water-scavenging effect of the nanofillers, previously reported in similar systems designed with PEO [121] in lithium batteries. Hence, the overviewed hybrid systems are in sight of being the most effective approach for improving the performance of solid-state electrolytes. 

## 4. Nanocomposite Polymer Electrolytes Based on Other Synthetic Polymers

As discussed, the development of electrochemical devices that make use of polymer electrolytes has gotten considerable interest. It is currently developing PEs with sufficiently high room temperature conductivity. The choice of the polymer is then known to depend principally on the presence of polar groups with sufficient electron donor power to form coordination with cations and a low hindrance to bond rotation [122], besides biodegradability and recyclability. Some synthetic polymers have been successfully used as a host material to prepare PEs for specific applications (Table 5). Few studies in the field have developed electrolytes with these polymers. However, the results presented left a precedent that deserves to be discussed for later studies that imply their use (Table 6). 

Poly(methyl methacrylate) (PMMA) has been the focus of a few studies due to its beneficial effects on the stabilization of the electrode-electrolyte interface [136]. PMMA is non-biodegradable, and 100% recyclable [137]. Nevertheless, its recycling process is not environmentally viable due to the produced harmful products, limiting its use [138]. PMMA based GPEs happen to present very high transparency in the visible region. Furthermore, they present the ability to be diluted in various organic solvents [139]. However, they show poor dimensional stability. Although they appear solid-like, they exhibit flow properties. Poor mechanical properties offset a good conductivity achieved of such plasticized film at a high concentration of the plasticizer [140]. To overcome the drawbacks presented by PMMA film, it has been blended with other polymers to improve the segmental motion in polymer hybrid systems and hence a more flexible and elastic material.

Sarojini et al. [124] developed a blended polymer matrix of PMMA and PVdF for magnesium cells. It also included ethylene carbonate as a plasticizer, Mg(Tf)_2_ as ionic salt and MgO as nanofiller. The best ionic conductivity increased the value by five orders of magnitude (~10^−6^ S·cm^−1^). This result was obtained thanks to the addition of the nanofiller, causing high conduction pathways. Nevertheless, the result was deficient to be considered for any application.

A blended polymer matrix composed of PMMA and PVdF-co-HFP was developed by Mishra et al. [127] for the design of an NCPE system for Zn cells. XRD and SEM studies confirmed the desirable amorphous and porous structure for the electrolyte. The best ionic conductivity, 4.3 × 10^−3^ S·cm^−1^, was obtained with 2 wt % of SiO_2_. The conductivity variation for these films obeyed the behavior of having two maxima, previously reported in other works [81,101]. A proton battery was assembled with the electrolyte, employing Zn/ZnSO_4_·7H_2_O as anode and PbO_2_/V_2_O_5_ as the cathode. The OCV for the battery was found at 1.55V. Besides, it showed rechargeability up to three cycles, and afterward, its discharge capacity faded away substantially. 

Poly(ethyl methacrylate) (PEMA) is a very similar material to PMMA but with a lower T_g_ and has been shown to possess higher mechanical strength than PMMA [141]. Besides, PEMA shows excellent chemical and high surface resistance. In addition, it offers high optical transparency [142], a property that could be desired for devices where the electrolyte is located in a visible region of the device. PEMA was employed for an NCPE in work [125], along with magnesium triflate and 1-ethyl-3-methylimidazolium bis(trifluoromethylsulfonyl) imide (EMITFSI), dispersed with MgO for Mg cell electrolytes. SEM analysis confirmed the obtention of the amorphous nature of the films. In addition, TGA curves revealed that the more significant amount of MgO in NCPE slowed down the mass loss rate of decomposition products. However, the electrochemical potential window for the highest conducting sample assumed that magnesium ion was not predominantly the factor to the ionic conductivity enhancement of NCPE. 

A blended polymer matrix was developed with PEMA and poly(vinyl chloride) (PVC), plasticized with zinc triflate, and EMIMTFSI ionic liquid was added for a novel NCPE by Candhadai et al. [128]. After that, it was doped with fumed SiO_2_ as a nanofiller. This film exhibited the highest ionic conductivity value of 6.71 × 10^−4^ S·cm^−1^ for a 3 et al SiO_2_. The increment of the amorphous phase was confirmed by XRD analysis. It resulted in slight progress in the zinc ion transport number and a wide ESW of ~5.07 V. This value ensured feasible zinc stripping/plating in the redox process involved. TG and DSC ascertained the improved thermal stability up to 180 °C and the reduction in T_g_. The exact blend, PVC and PEMA, was filled with nano-sized fillers Al_2_O_3_, TiO_2_ in the report by Prasanna et al. [129] for a zinc rechargeable battery. A high transport number value of 0.67 was obtained. From the studies analyzing glass transition temperature, the addition of fillers attenuated the values obtained, effect understood in terms of the obstruction of the polymer chains by the formation of cross-linking centers due to the interaction between the Lewis acid groups of the ceramic particles and the polar groups of the polymer chains. 

**Figure 12:**
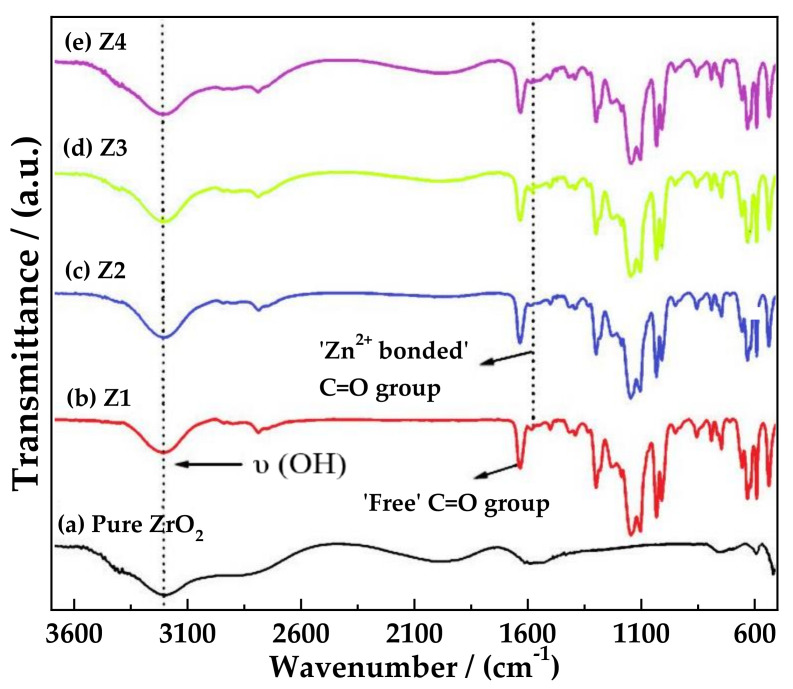
Room temperature ATR-FT-IR spectra of (**a**) pure ZrO_2_ (**b**–**e**) NCPEs with varying concentrations of ZrO_2_ in the wavenumber ranging from 4000 to 400 cm^−1^ at room temperature. Reproduced with permission from Polym. Compos., 40, Sai Prassana et al., PVC/PEMA-based blended nanocomposite gel polymer electrolytes plasticized with room temperature ionic liquid and dispersed with nano-ZrO_2_ for zinc ion batteries, 3402–3411, 2019 [130].

Based on the previous work, Prasanna et al. [130] continued the research by changing the nanofiller employed, being zirconia (ZrO_2_) the object of the study. The zinc ion transference number of 0.66 was almost the same obtained before with Al_2_O_3_ and TiO_2_. DSC and TG analysis confirmed the improved thermal behavior of ZrO_2_ added GPE compared to that of filler-free gel electrolytes. The interaction and complexation of the polymer components were probed by ATR-FT-IR analysis (Figure 12). The amplified coordination of Zn^2+^ cations and ceramic phase with C=O group was evidenced by the existence of a peak at 1721 cm^−1^ ascribed to the C=O group of PEMA in Figure 12b–e, upon the addition of 1 wt % nanofiller. The oxygen atoms of C=O group in PEMA generally acts as an electron donor resulting in the formation of a coordinate bond with zinc ions, and the addition of fillers enhances the intensity of this band, through hydrogen bonding between carbonyl oxygen (C=O) and the hydroxyl surface group (Zr-OH) of ZrO_2_ thus forming -Zr-O…H…O=C- species. Ultimately, it was observed better thermal stability up to 180 °C, and ESW to 3.87 V. 

These authors, in another work, incorporated the use of nano-sized tin oxide (SnO_2_) [131]. XRD and SEM studies were performed, confirming the existence of porous morphologies. Furthermore, the dispersion of SnO_2_ improved the thermal behavior of the composite system to 185 °C, which was ascertained by TG analysis. The ESW was found to be 4.37 V. Together with a feasible zinc plating/stripping process of the gel composite sample, these features implied good potential applicability of such films as electrolytes.

Poly(vinylpyrrolidone) (PVP) is a biocompatible polymer. It is a virtually non-biodegradable polymeric lactam with an internal amide bond. The tertiary amide carbonyl groups of PVP present a Lewis base character such that PVP can form a variety of complexes with a wide range of inorganic salts [143]. It is also hygroscopic and easily soluble in water and organic solvents such as alcohol. It presents a high T_g_ of 170 °C, because of the rigid pyrrolidone group. However, water can be employed as a plasticizer lowering this value to below 40 °C [144]. Besides, it is inert, shows good environmental stability, easy processing, excellent transparency, and a strong tendency for complex formation with smaller molecules [145]. This polymer is studied because of its thermal stability and cross-linked composites having high mechanical strength. It also has good mechanical and electrical characteristics. 

**Figure 13:**
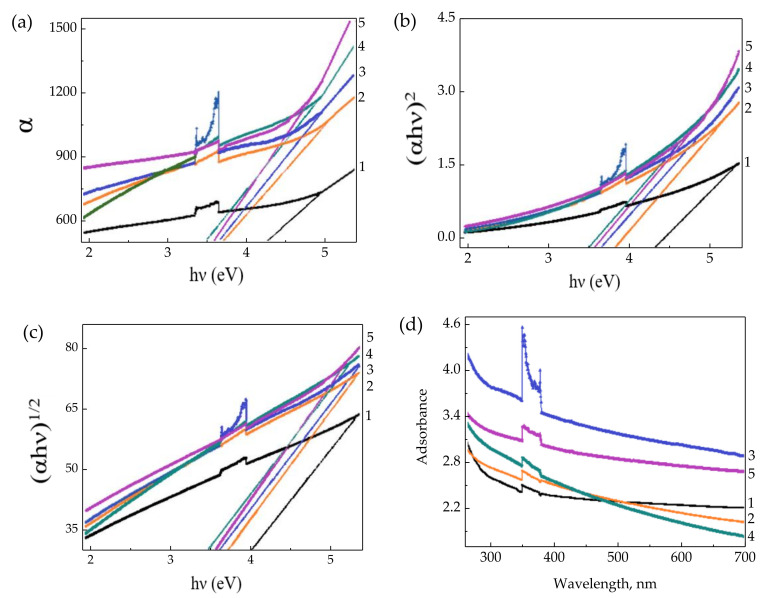
(**a**) hν vs α plots, (**b**) hν vs (αhν)^2^ (×10^7^) plots and (**c**) hν vs (αhν)^1/2^ plots and (**d**) UV–Vis spectra of polymer electrolyte films for different wt % ratios of pure PVP and polymer electrolytes: (1) pure PVP, (2) (95:5), (3) (90:10), (4) (85:15), (5) (80:20), Reproduced with permission from Polym. Sci.—Ser. A., 59, Shahenoor Basha et al., Optical and dielectric properties of PVP based composite polymer electrolyte films. 554–565, 2017 [126].

Basha et al. [126] developed an SPE composed of PVP and MgCl_2_x6H_2_O, with the addition of Al_2_O_3_ particles. Structural analysis showed orthorhombic lattice as evidence of a semi-crystalline nature present in the films. Optical analysis was used to identify the optical band gap of the material in the transmitting radiation. Graphs were plotted between absorption coefficient α, (αhν)^2^ and (αhν)^1/2^ as a function of hν (Figure 13a–c) to calculate bandgap energy values. The optical properties revealed that for the composition of 15%, the bandgap energy was the lowest among all weight ratios. Hence, it was obtained that the films with the lowest activation energy had the highest conductivity. UV–Vis spectroscopy was performed in the 300–700 nm (Figure 13d). This tool showed to be helpful for the identification of intra molecular vibrations of inorganic complexes in solution. Two spectral peaks are observed at 350 nm, which is due to the π–π* transition. Besides, a small peak was at 425 nm, correlated with the benzene and quinone rings in the polymer chain. 

**Figure 14:**
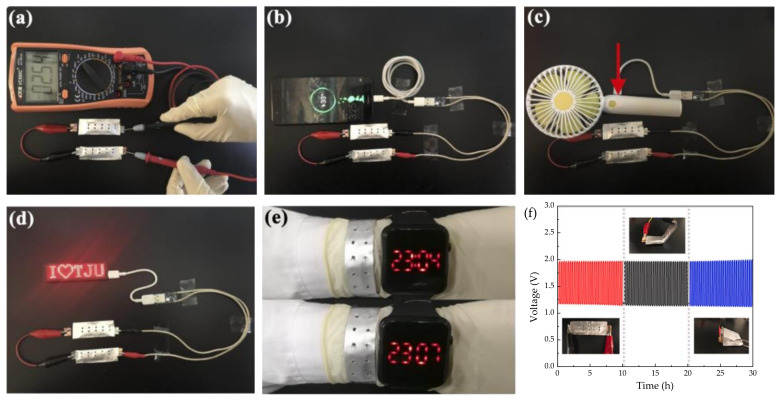
(**a**) Open circuit potential demonstration with two ZABs in series. A demonstration of (**b**) a mobile phone, (**c**) a handheld electric fan, and (**d**) an LED screen powered by two ZAB sets. (**e**) Photographs of an LED watch powered by a fabricated bracelet-type ZAB. (**f**) GCD curves of the ZAB under different bending conditions with corresponding photographs. Reproduced with permission from Nano Energy, 56, Fan et al., Porous nanocomposite gel polymer electrolyte with high ionic conductivity and superior electrolyte retention capability for long-cycle-life flexible zinc–air batteries, 454–462, 2019 [134].

Poly(vinyl alcohol) is a semi-crystalline synthetic biodegradable polymer from petroleum sources that presents various hydrophilic functional hydroxyl groups, which can favor water absorption. As a result, it shows a very high dielectric strength (>1000 kV mm^−1^), good charge storage capacity, good mechanical properties, high tensile strength, abrasion resistance, and dopant-dependent electrical and optical properties [146]. In addition, PVA has several advantages, such as high hydrophilicity, high gel strength, nontoxicity, and low cost [147]. Fan et al. [134] designed a zinc-air battery (ZAB) assembled with a semi-solid/solid-state electrolyte constructed with PVA and the optimum addition of SiO_2_. The ZAB presented excellent cycling stability over 48 h, stable discharge performance, and relatively high-power output. Flexibility was an outstanding feature obtained with no degradation through bending conditions. In the results, this cell was able to power a handheld electric fan, a light-emitting diode screen, or even a mobile phone (Figure 14), showing its promising potential for high-performance ZABs along with high safety, cost-effectiveness, excellent flexibility, electrolyte retention capability, as well as good thermal and mechanical properties.

The PVA electrolytes filled with nano ZnO transport parameters were conducted by Abdullah et al. [135] using the Rice–Roth model for proton-conducting batteries, explaining that the moderate addition of nanofiller enhances ionic conductivity by increasing mobility and number density of mobile proton ions. Another ZAB was designed to implement MWCNTs into the electrodes by Wang et al. [148], to improve the performance of the cell. It was found that MWCNTs were effective conductive additives in the anode as they bridged the zinc particles. The electrolyte was composed of poly(acrylic acid) (PAA) and PVA. A limitation for this NCPE was water evaporation because of the volatility character of the films. 

Poly(ε-caprolactone) (PCL) is a biodegradable polymer that is nontoxic and widely used in biomedical applications because of its considerable degradation time in an aqueous medium and contact with microorganisms [149]. It is a synthetic thermoplastic polymer derived from crude oil and synthesized through the polymerization of ε-caprolactone monomer by a stannous octanoate catalyzed ring-opening mechanism [150]. It presents good mechanical properties [151]. Furthermore, it is a candidate polymer host for ionic conduction because it contains a Lewis base (ester oxygen) that can coordinate cations due to carbonyl functional groups in its backbone structure. 

PCL was doped with zinc triflate and octadecylamine modified montmorillonite (ODAMMT) nano clay by Sownthari et al. [132]. The maximum electrical conductivity was 9.5 × 10^−5^ S·cm^−1^ for 15 wt % loadings of nano clay into the polymer-salt complex. XRD and DSC analysis confirmed the decrease in crystallinity. The electrolyte degradation happened in 90 days, making this electrolyte a promising candidate for battery applications. 

An optimized NCPE composed of PCL, zinc triflate and the incorporation of Al_2_O_3_ was prepared by Sownthari et al. [133]. The complexation of polymer, salt, and filler was confirmed from FT-IR studies. The various relaxation processes associated with the conductivity mechanism were also analyzed during the investigation. From this, it was revealed that the chain length of polymer PCL was so long that the bond rotation was favorable only at low frequency, so the filler increased the amorphicity within the polymer network, and thus the rotation becomes feasible, making a shift toward higher frequency side which meant a shorter relaxation time. The increase of conductivity was mainly due to an apparent rise in the number density of charge carriers which was confirmed from FT-IR and dielectric studies. The increasing trend of dielectric constant matched well with the conductivity variation as a function of filler concentration.

## 5. Nanocomposite Polymer Electrolytes Based on Biopolymers

Most synthetic polymers are detrimental to the environment because of their non-biodegradability [152]. Consequently, the application of biodegradable polymers in energy storage devices is currently paramount in designing the next generation of batteries to reduce environmental impact. For a biobased polymer to be considered ecological, its origin and production technique are also of importance. Cellulose, starch, chitosan, agar, and carrageenan are some of the most common polymers used as hosts for batteries [51] (Table 7). 

Among the wide range of applications available for batteries, there is a need to design biocompatible batteries for implants that need a power source to perform their functions in the biomedical field. They go from sensing or stimulation to influencing critical biological processes like wound healing, tissue regeneration, or brain activity. Unfortunately, little work has been done so far to develop bioresorbable electronics or self-deployable power sources [153,154,155]. However, the present review pretends to show the promising results obtained so far with electrolytes that, with some modifications, could be employed in the biomedical field. Table 8 summarizes the features presented by these NCPEs.

### 5.1. Cellulose

Cellulose is a biopolymer known to be the most abundant polymer in nature. It presents a molecular weight ranging from 300,000 to 500,000 Da. Its molecule offers three hydroxyls groups that can be modified to make the molecule water-soluble [176]. Many cellulose derivatives can be obtained from this modification, classified between cellulose ethers and cellulose esters. Cellulose has been applied in batteries for electrodes or separators as GPEs [176] and as binders/surface modifiers for graphite anodes for batteries. Moreover, synthetic polymers often need high-temperature processing stages to prepare GPEs, while with nanofiber cellulose (NFC) hydrogels, they can be cured at temperatures close to ambient temperature [177]. 

Cellulose was never intensively used as a polymer electrolyte in advanced batteries until Johari et al. [157] reported an NCPE based on cellulose acetate dispersed with SiO_2_ for a battery with the configuration Zn/composite cellulose electrolytes/MnO_2_. The results showed the expected increase in ionic conductivity and an OCV of 1.6V. The constancy of the assembled cell was tested for 24 hours. However, no further electrochemical studies were performed. In a posterior work [158], the same authors reported an NCPE based on cellulose acetate dispersed with nanosized TiO_2_ particles for a battery with the same composition as the previous one. The OCV characteristic of the cell at room temperature showed that the initial voltage of the cell is 1.55 V, dropping to 1.40 V within the first two hours of assembly. The cell voltage was observed to have stabilized at this voltage, and the OCV remained constant at 1.40 V for a period of 24 h. The fabricated cell was reasonably stable in the open cell condition.

An NFC hydrogel was synthesized by Poosapati et al. [156] by adding gelatine, polyacrylic acid (PAA), and potassium hydroxide (KOH) as additives. The hydrogel with the most appropriate amounts of additives got an ionic conductivity of 0.1 S·cm^−1^, representing an increase of five orders of magnitude from the pristine hydrogel. This report concluded that the small amounts of additive present helped enhance the mechanical stability and ionic conductivity by changing the degree of crystallinity and ionic concentration in the hydrogel layers.

Another approach for cellulose applications has been made by employing it as a soaked separator electrolyte. Zhang et al. [178] studied a functionalized with quaternary ammonium (QA) laminate-structured nanocellulose/GO membrane, developed for a hydroxide-conducting electrolyte for zinc-air batteries. Herein, cellulose was utilized to interconnect the framework to integrate GO into a flexible membrane with higher water content. Achieving a laminated cross-linked structure eliminated the risk of pushing water out of the membrane when handling or bending, besides good adhesion to the electrodes. The membrane’s enlarged d-spacing enhanced the mobility of hydroxide ions by vehicle mechanism, besides its lower activation energy. Water molecules also could have caused mobility by the Grotthus mechanism. At 70 °C, the ionic conductivity of 0.0588 S·cm^−1^ and an OCV of 1.4 V was achieved.

### 5.2. Chitosan

Chitosan is environmentally friendly and an excellent membrane-forming polymer material. It is known for being non-toxic, biodegradable, and biocompatible, making it a good solution for many electrochemical applications that can be modified to get electrolytes. Chitosan is produced from the deacetylation reaction of chitin. Chitin is a natural polysaccharide generally found in the exoskeleton of arthropods and various fungi [179]. Chitosan is widely applied in lots of fields, as in biotechnology, biomedicine [180]. Its molecule presents several polar groups, such as hydroxyl and amino groups, forming complexes with inorganic salts. However, pristine chitosan shows a very low ionic conductivity (10^−9^ S·cm^−1^) [181], the fact that it is tried to be enhanced by the addition of salts and fillers. Very little work has been done for chitosan NCPE applied in zinc or magnesium batteries. However, some authors have endeavored to implement nanofillers for lithium electrolyte applications [160,182,183,184,185,186,187,188], so the most noteworthy ones are now discussed.

Hexanoyl chitosan was employed as a polymer matrix with TiO_2_ as filler and lithium perchlorate (LiClO_4_) as doping salt to design NCPEs by Muhammad et al. [159]. The electrolyte system was characterized by impedance spectroscopy. It was shown that the increment in conductivity was caused by the increase in the mobility of free ions and the increase in the free ion concentration. The XRD results obtained for this electrolyte in a posterior work of the authors [189] confirmed the decrease in crystallinity, leading to the expected increase in conductivity that was modeled by the Rice and Roth model. 

A system of the same composition based on hexanoyl chitosan + LiClO_4_ + TiO_2_ was reported by Winie et al. [190], who reported the complexation of the polymer and the salt as a result of the shift of N(COR_2_), O=C-NHR, and OCOR bands of hexanoyl chitosan to lower wavenumbers, and supported the use of chitosan as a polymer host in terms of the presence of lone pair electrons at the nitrogen and oxygen atoms where inorganic salts can be solvated. Results showed that both dielectric constant and dielectric loss decreased with increased frequency and increased with increased temperature.

Aziz et al. [162] reported novel chitosan-ammonium thiocyanate (NH_4_SCN) complexes doped with nanosized Al_2_O_3_ filler. FT-IR and XRD confirmed the complexation between the cation of the salt and the donor atom in chitosan polymer. The high filler content increased the T_g_ value since it increased the crystallinity of the sample, as depicted by XRD. Alumina was employed in chitosan and lithium triflate (Li(Tf)_2_) [191], where the AC conductivity studies showed the promising features already told for this kind of biopolymer system.

In a work by Navaratnam et al. [163] chitosan was used as the host polymer in a designed system consisting on LiCF_3_SO_3_ as the dopant salt, EC and PC as the plasticizers, and different concentrations of SiO_2_ as the inorganic filler. The obtained ionic conductivity for this system was very low to consider for practical applications. However, in a following article by Rosli et al. [161], it was presented a study comparing the type of filler and their effect on the electrical properties in the polymer electrolyte, resulting in a higher conductivity enhancement brought about by TiO_2_ compared to SiO_2_ for the system hexanoyl chitosan-LiClO_4_ polymer electrolyte. This finding can be understood by the more acidic nature of TiO_2_, which promoted a greater degree of salt dissociation. Zirconia was employed as a nanofiller for the previous electrolyte composition of LiClO_4_ as salt and chitosan by Sudaryanto et al. [164], and the films were characterized by XRD and EIS. The obtained results were quite comparable to the previously discussed work since the ionic conductivity was slightly higher. Besides, the obtained ion transference number of 0.55 was considered quite enough to apply it in an ion battery. 

For magnesium batteries, a GPE based on chitosan, magnesium triflate, and EMITf was developed by Wang et al. [192]. The results showed that the Mg-ion mechanism was the complexation and decomplexation of Mg^2+^ with amine band (NH_2_) from chitosan. The relaxation time of the electrolyte membrane was as low as 1.25 × 10^−6^ s, indicating that the mobility of ions was relatively high. The electrochemical properties of this GPE, presented in Table 7, were considered a precedent for future practical applications, and some latest reports were reported until the present date for chitosan polymer electrolytes for EDLC devices [193,194,195]. Still, they are out of the scope of this review. To the best of our knowledge, no studies are reporting NCPEs made of chitosan for magnesium batteries.

### 5.3. Starch

Starch is one of the most popular renewables and biodegradable polymers found as granules in plants. It is composed of a mixture of linear amylose (α(1,4) linked anhydroglucose) and branched amylopectin (α(1,6) linked anhydroglucose) polysaccharide chains. At the same time, it can undergo derivatization reactions. It is introduced some functional groups into the starch molecule, resulting in the alteration of its gelatinization, pasting, and retrogradation behavior [196]. Starch is known to be abundant in nature due to its wide variety of sources, consisting of several kinds of food plants from which it comes. Hence, besides its application in the food industry, it is applied industrially as binders and adhesives, absorbents and encapsulants, as coatings and sizes on paper, textiles, and carpets [197]. In addition, efforts have been made to use starch to create thermoplastic materials [198,199]. 

**Figure 15:**
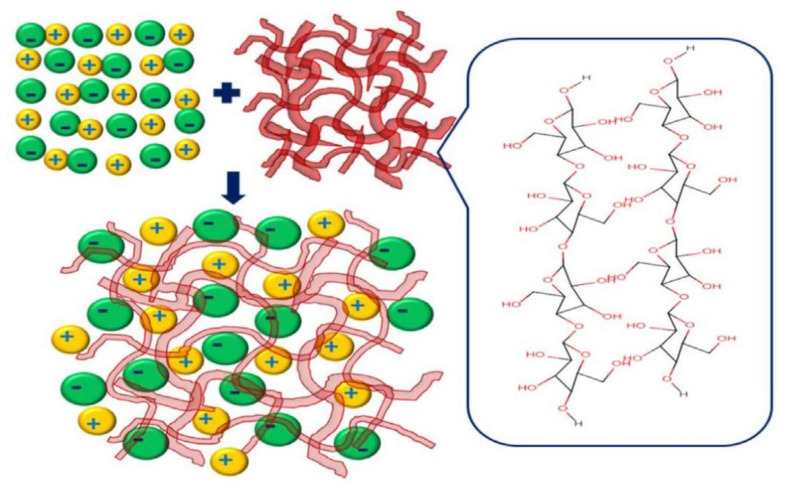
Schematic diagram showing ion dissociation in biopolymer salt matrix. Reproduced with permission from Renew. Sustain. Energy Rev., 65, Singh et al., Perspectives for solid biopolymer electrolytes in dye sensitized solar cell and battery application, 1098–1117, 2016 [50].

In terms of application for magnesium batteries, potato starch was doped with magnesium acetate (Mg(C_2_H_3_O_2_)_2_) [165], and the effect of glycerol and 1-butyl-3-methylimidazolium chloride (BmImCl) was studied in terms of conductivity and dielectric properties. It was concluded that too much plasticizer causes the salt to recrystallize, causing the cations to hardly coordinate at the polar atoms, decreasing the ionic conductivity. The effect of salt concentration in the biopolymer electrolyte matrix is demonstrated through dissociated ions model (Figure 15). Rice starch was employed for an NCPE composed of lithium iodide (LiI), 1-methyl-3-propylimidazolium iodide (MPII) as an ionic liquid, and TiO_2_ nanopowder by Khanmirzaei et al. [166]. The resulting electrolyte was employed to build a DSSC, showing an efficiency of 0.17 at 1000 W·m^−2^ light intensity.

From the variety of sources available for starch, a biodegradable corn starch–lithium perchlorate (LiClO_4_)-based SPE with the addition of nano-sized fumed silica [167] was prepared by solution casting technique. FT-IR results confirmed some complexation between corn starch, LiClO_4_, and silica. Excessive SiO_2_ content decreased the ionic conductivity through agglomeration of particles and cross-linking in the polymer, showed by DSC, TGA, and SEM studies. The same investigation group reported the NCPE applied in electric double-layer capacitors (EDLCs) [168]. The device was characterized by CV, galvanostatic charge-discharge, and AC impedance spectroscopy. The discharge characteristics were almost linear, which confirmed the capacitive behavior of the EDLC cell. The fabricated EDLC cells performed good cyclability up to 500 cycles with more than 90% coulombic efficiency.

A solid electrolyte designed by blending chitosan with corn starch for application in an electrochemical double-layer capacitor (EDLC) and proton batteries was reported [169]. From transference number measurements (TNM), the electrolytes’ transference number of ion (t_ion_) showed that ion is the dominant conducting species. The transference number of cation (t_+_) for the highest conducting electrolyte was found to be 0.56. Linear sweep voltammetry (LSV) result confirmed the suitability of the highest conducting electrolyte to be used to fabricate EDLC and proton batteries. The open-circuit potential (OCP) of the primary proton batteries for 48 h was lasted at (1.54 ± 0.02) V, while that of secondary proton batteries lasted at (1.58 ± 0.01) V. 

Masri et al. [170] used sago powder (starch from various tropical palms) to design a sago-KOH GPE. Then, it was employed in an experimental Zn-air battery using a porous Zn electrode as the anode. The battery showed outstanding discharge capacity and practical capacity obtained of 505 mAh·g^−1^. In parallel, Zahid et al. [200] designed a GPE for zinc-air batteries based on cassava (Manihot esculenta), one of the most essential starch sources in tropical and subtropical areas. The highest ionic conductivity obtained was 4.34 × 10^−3^ S·cm^−1^.

### 5.4. Carrageenan

Carrageenan is a linear sulfated polysaccharide polymer extracted from a marine red seaweed called Rhodophyceae and *Kappaphycus alvarezii*. It is consisted of repeating units of (1,3)-d-glucopyranose and (1,4)-3,6-anhydro-α-d-glucopyranose [201]. Besides, based on the number of sulfate groups, it is classified into three types: Kappa (κ)-carrageenan (one sulfate per disaccharide), iota (ι)-carrageenan (two sulfates per disaccharide, and lambda (λ)-carrageenan (three sulfates per disaccharide). Carrageenan is hydrophilic due to the presence of hydroxyl groups and the mentioned sulfate groups in it. In terms of electrochemical properties, this polymer is known for being in rich hydroxyl groups and oxygen atoms which are essential for interaction and coordination with cations [202]. Carrageenan has been used in various applications, including food, pharmaceutical, and cosmetic industries as viscosity builders, gelling agents, and stabilizers [203], even proving to have anti-tumor and anti-angiogenic activity [204], besides being applied in drug delivery systems and other biomedical applications [205]. In this field, Sabbagh et al. [206] have investigated the nanocomposite positive effects on structural, functional, morphological, and thermal properties of carrageenan hydrogels, obtaining promising results for drug-delivery systems. Moreover, it is known that carrageenan could be used as a prominent electrolyte in electrochemical devices with suitable modifications.

**Figure 16:**
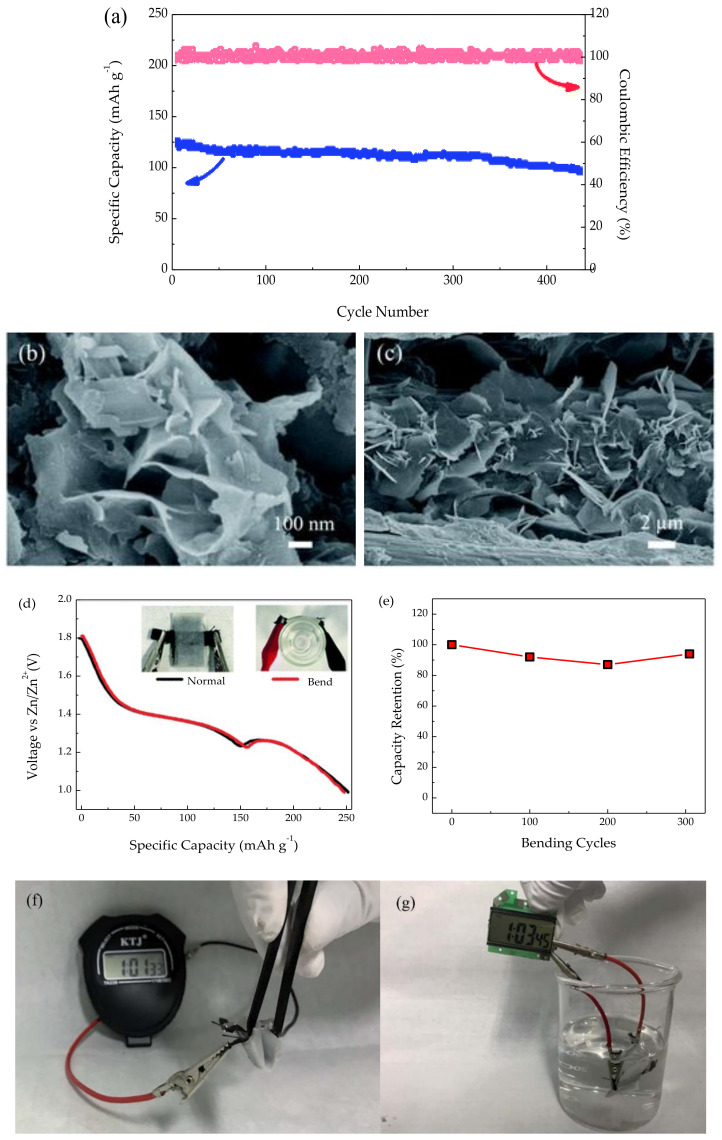
(**a**) Cycling stability of the solid-state ZIBs with KCR electrolyte cycled at 6.0 A·g^−1^ and corresponding coulombic efficiency. SEM images of (**b**) the MnO_2_ cathode and (**c**) the electroplated Zn anode after 450 charge/discharge cycles. (**d**) Discharge curves under normal and bending conditions. (**e**) The bending test of solid-state ZIBs with KCR electrolyte for 300 cycles. (**f**) A solid-state ZIB with KCR electrolyte powers a timer under 180 degrees of bending conditions. (**g**) A solid-state ZIB with KCR electrolyte powers a timer when the battery is fully immersed in the water. Reproduced with permission from RSC Adv., 9, Flexible quasi-solid-state zinc ion batteries enabled by highly conductive carrageenan bio-polymer electrolyte, 16313–16319, 2019 [171].

A rechargeable quasi-solid-state zinc ion battery using κ-carrageenan bio-polymer electrolyte was reported by Huang et al. [171]. The mechanical robustness of the electrolyte was reinforced by using a rice paper scaffold, which reduced the chances of short circuits as well. The κ-carrageenan electrolyte was found to be highly conductive. Furthermore, electrolyte production did not need water and oxygen-free environment or other protection measures, which is ideal for scaling up production. The zinc ion battery assembled with this biopolymer electrolyte also showed excellent cycling stability; 80% of its initial capacity still remained even the cyclic number extended to 450 cycles at 6.0 A·g^−1^ (Figure 16a). The morphology of cathode and anode materials remained after 450 cycles, almost unchanged compared to the morphology of the pristine MnO_2_ and Zn (Figure 16b,c). Experimental results showed that the batteries maintained the discharge profile and AC impedance spectra after the test (Figure 16d). Besides, after 300 bending cycles, 95% capacity was retained (Figure 16e). This work brought new research opportunities in developing low-cost, flexible solid-state zinc ion batteries using green natural polymer, besides being capable of powering a timer under bending condition (Figure 16f), and also powered a timer when the entire device was immersed in water, demonstrating its good waterproofness (Figure 16g). 

For magnesium ion conduction, an SPE consisted of κ-carrageenan with MgCl_2_ salt was designed by Sangeetha et al. [172], employing it in a primary magnesium battery. The resulting OCV for the battery was 2.17 V. In another report, κ-carrageenan containing tri-iodide/iodide redox couple was modified for dye-sensitized solar cell applications with nanofillers such as TiO_2_, iron (III) oxide Fe_2_O_3_, and halloysite by Chan et al. [207]. For this system, the addition of various fillers to the PE system increased the dissociation of iodide ions and improved the ionic conductivity of the cells. DSSC characterization revealed a low efficiency due to relatively high charge transfer resistances at the TiO_2_/dye/electrolyte interface.

### 5.5. Agar

Agar is defined as a strong gelling hydrocolloid from marine algae. It is constituted by repetitive units of D-galactose and 3,6-anhydro-L-galactose, with few variations and low content of sulfate esters [208]. Agar is widely known because of its gelling power, based exclusively on the hydrogen bonds formed among its linear galactan chains, providing excellent reversibility. Its unique properties make it suitable for many applications, especially in preparing microbiological culture media [209]. It is widely used in the food industry, in cosmetics, and in microbiology [210]. In terms of electrochemical properties, agar forms a slightly viscous solution on dissolving in hot water and then turns into a thermo-reversible gel when cooled down. Agar attracts attention because of its best film-forming capability, used in synthesizing agar hydrogel used as an electrode binder in fuel cells [211]. 

A novel PE based on agar and doped with NH_4_SCN was prepared for zinc cells by Selvalakshmi et al. [173]. The obtained results ensured that it is a good candidate for a low-cost biopolymer electrolyte membrane for fuel cell applications and solid-state devices. Similarly, Alves et al. [174] prepared a PE-based on agar doped with magnesium triflate for magnesium ion conduction, but the obtained results were not suitable for practical applications. 

In terms of the implementation of nanofillers, some studies of agar applied in electrolytes for dye-sensitized solar cells have been presented. A polysaccharide GPE composed of agar in 1-methyl-2-pyrrolidinone (NMP) as a polymer matrix, LiI)/iodine (I_2_) as a redox couple, and TiO_2_ nanoparticles as fillers were reported by Yang et al. [212]. Results showed that optimizing the electrolyte composition, such as agar and TiO_2_ concentration, is necessary to improve the energy conversion efficiency of the DSSCs. Likewise, similar results were obtained for the same electrolyte system by Wang et al. [175], where the effects of LiI concentration were analyzed.

All the presented results so far show that NCPEs based on biopolymers solve the issues presented by all the kinds of PEs (increasing the low ionic conductivities and improving the mechanical properties). Furthermore, nanofillers have been shown to help prevent the dissolution of the salts provoked by the polar groups presented in the polysaccharide structure of most of the studied biopolymers, enhancing the ionic conductivity. Moreover, in contrast to the synthetic polymers mentioned in the previous sections, the presence of a wide variety of functional groups in biopolymers make them capable of showing various kinds of bonds and intermolecular forces. This feature is of vast importance in terms of enhancing the mechanical stability of the matrix. Nevertheless, to confirm the benefits of these kinds of forces, more attention to mechanical testing is recommended to determine the best application for these matrixes after paying attention to the alternates of material processing available so far.

## 6. Conclusions

According to the literature available so far, the key aspects related to nanocomposite polymer electrolytes for batteries composed of zinc or magnesium have been presented and discussed along with the review to know their suitability for application in rechargeable cells.

The copolymer matrix composed of PVDF-co-HFP has been shown to hold on to the ionic liquid and retain it in the membranes. Moreover, according to the results by the number of publications so far, the improved results in conductivity obtained for this matrix along with the addition of nanofillers, provoke a space-charge region, understood by the evistence of free electrons at the surface of the nanocomposite, facilitating the new kinetic path for ionic transport and polymer segmental motion. This mechanism ensures the electrolyte to be capable of ion transference. Improved high ionic conductivity and better thermal and mechanical stability compared to liquid electrolyte systems have been confirmed. Moreover, when a specific percentage of nanofiller is added, a decrease in ionic conductivity is observed. Excessive fillers could provoke this in the NCPE that may trigger the formation of ion pairs and ion aggregation, such as the non-conducting phase presented as an electrically inert component blocking ion transport. 

By adding nanoparticles, it has been possible to reduce the degree of crystallinity of the polymers, an aspect proven in the investigations on PEO electrolytes. This feature is a crucial issue because the membrane’s amorphous degree is responsible for conduction along with the electrolyte. Furthermore, conductivity is improved because the nanoparticles can act as a solid plasticizer, ensuring electrochemical properties and mechanical strength. Besides, it is discussed the NCPEs assembled with other synthetic polymers. Despite some minor variations, the results presented replicate the argument of improving the electrolyte properties by adding nanofillers, setting a solid precedent regarding the applicability of these polymers, where not much research has been found with the discussed approach.

Biopolymer electrolytes based on natural molecules have shown comparable ion conduction and electrochemical properties with traditional synthetic polymer electrolytes as the ones discussed in previous sections. Besides, when discussing stability, the natural polymer-based electrolytes are comparable, if not better, than the synthetic ones. The processes for enhancing their properties are reachable by the same methods. The conduction mechanism in both electrolytes is the same and is explained in terms of the exchange of ions between complexed sites. Moreover, their abundance, low cost, and easier processing ability make biopolymer electrolytes expected to bring a better future of green technologies than non-biodegradable, toxic, and harmful materials used in commercial batteries today. 

When choosing which state is better for the electrolyte, between making it a solid or a gel, it is paramount to consider the device’s application. Gel polymer electrolytes have been shown to be capable of being employed in conditions where flexibility is well appreciated. However, GPEs currently need mobile liquids to perform the conduction process, and the current ones present concerns in terms of stability, safety, and general sustainability. Hence, the search for more benign and environmentally-friendly mobile liquids is a current issue to increase the expectation on developing batteries based on sustainable components. 

In general, for all the polymer electrolytes, the addition of nanoparticles (ZnO, MgO, TiO_2_, Al_2_O_3_, SnO_2_) has been proved to enhance the electrical conductivity by, in the least of the cases, one order of magnitude. Moreover, conductivity is improved, but cationic species’ electrochemical properties, mechanical strength, and transport properties are. Ultimately, the awareness of the addition of nanofillers improving the mechanical stability and ionic conductivity is a crucial point to be explored in the production of batteries. However, the state-of-the-art is still lacking in terms of the development of NCPEs based on biopolymers. The few investigations overviewed so far set a precedent for the demand for further research with this specific approach. These attempts need to be further developed to get practical applications for the industry in large scale of polymer-based electrolyte batteries, as well as other electrochemical devices, such as bioresorbable electronic devices that include biobatteries, offering an innovative solution to the problems currently faced by biomedical applications, generating positive impacts to the wellness of human beings and the environment. 

The research in this field needs to continue developing. Still, zinc and magnesium are absolutely the future of batteries that present electrolytes in solid-state. These metals are likely to replace lithium, thanks to their high energy potential, inherent safety, cost-effectiveness, and environmental-friendliness, along with the employment of the biodegradable biopolymers discussed in this article for the electrolyte. These features set the path for developing novel environment-friendly battery systems in the present world that urges for more sustainable options.

## Figures and Tables

**Table 1:** A summary of NCPEs composed of PVDF-co-HFP for magnesium batteries.

Nanocomposite	Ionic Salt	Conductivity (S·cm^−1^) 10^−3^	Activation Energy (eV)	Electrochemical Stability Window (V)	State	Reference
No added	Mg(Tf)_2_	0.15	-	5	Gel	[72]
	Mg(ClO_4_)_2_	0.293	0.33	4	Solid	[73]
SiO_2_	Mg(ClO_4_)_2_	3.2	-	4.3	Gel	[74]
	Mg(ClO_4_)_2_	11	-	3.5	Gel	[75]
	Mg(ClO_4_)_2_	10	-	3.5	Gel	[76]
Al_2_O_3_	Mg(Tf)_2_	3.3	-	3.3	Gel	[77]
MgAl_2_O_4_	Mg(Tf)_2_	4.0	-	3.3	Gel	[77]
Al_2_O_3_ *	Mg(NO_3_)_2_	0.101	-	-	Solid	[78]
MgO	Mg(ClO_4_)_2_	8	0.235	3.5	Gel	[79]
	Mg(ClO_4_)_2_	6	0.032	3.5	Gel	[75]
MgO *	Mg(NO_3_)_2_	0.104	0.45	-	Solid	[80]
MgO and SiO_2_	Mg(ClO_4_)_2_	10 and ∼9	-	-	Gel	[81]
ZnO	MgCl_2_	0.12	0.45	-	Solid	[82]
ZnO *	Mg(NO_3_)_2_	0.37	-	-	Solid	[83]
BaTiO_3_	Mg(Tf)_2_	0.411	-	-	Solid	[84]
TiO_2_ *	Mg(NO_3_)_2_	0.010	0.30	-	Solid	[85]

* PVDF without copolymerization with HFP.

**Table 2:** A summary of NCPEs composed of PVDF-co-HFP for zinc batteries.

Nanocomposite	Ionic Salt	Conductivity (S·cm^−1^) 10^−3^	Activation Energy (eV)	Electrochemical Stability Window (V)	State	Reference
No Added	Zn(Tf)_2_	1.73	0.025	-	Gel	[88]
	Zn(Tf)_2_	2.44 × 10^−2^	0.380	3.45	Solid	[89]
	Zn(Tf)_2_	0.144	-	4.14	Solid	[90]
TiO_2_	Zn(Tf)_2_	0.34	-	-	Solid	[91]
ZrO_2_	Zn(Tf)_2_	0.46	-	2.6	Solid	[92]
ZnO	Zn(Tf)_2_	6.7	-	4.5	Gel	[93]
CeO_2_SiO_2_	Zn(Tf)_2_	0.3	-	2.7	Solid	[57]
	NH_4_CF_3_SO_3_	1.07	-	-	Solid	[94]

**Table 3:** A summary of NCPEs composed of PEO for magnesium batteries in solid-state.

Nanocomposite	Ionic Salt	Conductivity (S·cm^−1^) 10^−3^	Activation Energy (eV)	Electrochemical Stability Window (V)	Reference
No added	Mg(Tf)_2_	56	0.49	4.6	[103]
	Mg(Tf)_2_	0.277	0.40	-	[101]
	Mg(ClO_4_)_2_	0.277	0.30	-	[104]
MgO	Mg(Tf)_2_	1.67	0.34	-	[101]
	(CH_3_COO)_2_Mg × 7H_2_O	363	0.013	7.6	[105]
	Mg(ClO_4_)_2_	1.04	0.29	-	[104]
TiO_2_	Mg(Tf)_2_	1.53	0.38	-	[101]
	Mg(ClO_4_)_2_	1.14	0.28	-	[104]
SiO_2_	Mg(Tf)_2_	0.586	0.36	-	[101]
	Mg(ClO_4_)_2_	0.87	0.28	-	[104]
B_2_O_3_	MgCl_2_	0.716	-	-	[106]
Starch nanocrystals (SNCs)	MgBr_2_	0.116	-	-	[107]

**Table 4:** A summary of NCPEs composed of PEO for zinc batteries in solid-state.

Nanocomposite	Ionic Salt	Conductivity (S·cm^−1^) 10^−3^	Activation Energy (eV)	Electrochemical Stability Window (V)	Reference
No added	ZnCl_2_	2.7 *	-	2.60	[109]
Al_2_O_3_	Zn(Tf)_2_	2.1	0.44	3.6	[110]
	Zn(Tf)_2_	~0.101	0.19	-	[111]
TiO_2_	ZnCl_2_	~100	0.087	-	[112,113]
SiO_2_	NH_4_HSO_4_	0.61	-	-	[114]
ZnO	Zn(Tf)_2_	0.184	0.23	-	[115]
Zn Fe_2_O_4_	NH_4_SCN	~10^−3^	-	-	[116]

* Results obtained at 55 °C.

**Table 5:** Chemical structures of some synthetic polymers employed in nanocomposite polymer electrolytes NCPEs.

Name	Structure	Glass Transition Temperature (T_g_) (°C) [123]
Poly(methyl methacrylate) (PMMA)	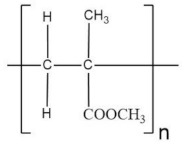	105
Poly(ethyl methacrylate) (PEMA)	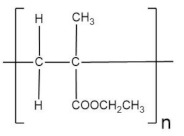	65
Poly(vinyl chloride) (PVC)	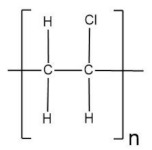	83
Poly(vinyl alcohol) (PVA)	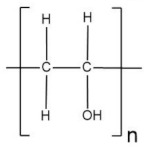	80
Poly(ε-caprolactone) (PCL)	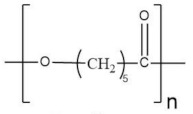	−66
Poly(vinylpyrrolidone) (PVP)	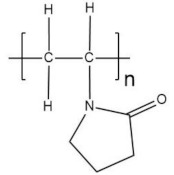	182

**Table 6:** NCPEs for magnesium and zinc batteries using other synthetic polymers.

Polymer	Nanocomposite	Ionic Salt	Conductivity (S·cm^−1^) 10^−3^	Activation Energy (eV)	Electrochemical Stability Window (V)	State	Reference
**Magnesium**							
PMMA + PVdF	MgO MgO Al_2_O_3_	Mg(Tf)_2_	1.29 × 10^−2^	-	-	Solid	[124]
PEMA		Mg(Tf)_2_	0.12	0.46	3.4	Solid	[125]
PVP		MgCl_2_ ⋅ 6H_2_O	1.22 × 10^−2^	-	-	Solid	[126]
**Zinc**							
PMMA/PVDF-co-HFP	SiO_2_	NH_4_SCN	43	0.196	3.2	Gel	[127]
PVC/PEMA	SiO_2_	Zn(Tf)_2_	6.71	-	5.07	Gel	[128]
	Al_2_O_3_ + TiO_2_	Zn(Tf)_2_	4.27	-	~4	Gel	[129]
	ZrO_2_	Zn(Tf)_2_	3.63	-	3.87	Gel	[130]
	SnO_2_	Zn(Tf)_2_	4.92	-	4.37	Gel	[131]
PCL	ODAMMT	Zn(Tf)_2_	0.95	0.46	4.5	Gel	[132]
	Al_2_O_3_	Zn(Tf)_2_	0.25	-	-	Gel	[133]
PVA	SiO_2_	-	5.73 × 10^2^	-	-	Gel	[134]
	ZnO	NH_4_NO_3_	4.71	0.92	-	Solid	[135]

**Table 7:** Chemical structures of some biopolymers employed in nanocomposite polymer electrolytes NCPEs.

Name	Structure	Glass Transition Temperature (T_g_) (°C) [123]
Agar	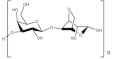	98
Carrageenan	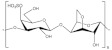	41
Cellulose	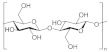	220
Chitosan	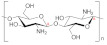	200
Starch	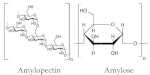	227

**Table 8:** Polymer electrolytes based on biopolymers for electrochemical applications.

Polymer	Nanocomposite	Ionic Salt	Conductivity (S·cm^−1^) 10^−3^	Activation Energy (eV)	Electrochemical Stability Window (V)	State	Reference
Cellulose (NFC)	No added	-	0.1	-	-	Hydrogel	[156]
Cellulose acetate	SiO_2_	NH_4_BF_4_	7.9 × 10^−3^	-	-	Gel	[157]
	TiO_2_	NH_4_BF_4_	1.4 × 10^−2^	0.12	-	Gel	[158]
Hexanoyl Chitosan	TiO_2_	LiClO_4_	3.06 × 10^−4^	-	-	Solid	[159,160]
	TiO_2_	LiClO_4_	3.1 × 10^−4^	0.08	-	Solid	[161]
	SiO_2_	LiClO_4_	1.96 × 10^−4^	0.12	-	Solid	[161]
	Al_2_O_3_	NH_4_SCN	5.86 × 10^−4^	-	-	Solid	[162]
Chitosan	SiO_2_	Li(Tf)_2_	4.38 × 10^−5^	0.26	-	Solid	[163]
	ZrO_2_	LiClO_4_	3.6 × 10^−4^	-	-	Solid	[164]
Potato Starch	No added	Mg(C_2_H_3_O_2_)_2_	1.12 × 10^−5^	-	-	Solid	[165]
Rice Starch	TiO_2_	LiI	3.6 × 10^−4^	0.22	-	Solid	[166]
Corn Starch	SiO_2_	LiClO_4_	1.23 × 10^−4^	0.25	3.0	Solid	[167,168]
Corn Starch/Chitosan	No added	NH_4_Cl	5.11 × 10^−4^	-	-	Solid	[169]
Sago (starch)	No added	KOH	4.45 × 10^−1^	-	-	Gel	[170]
κ-carrageenan	No added	-	3.32 × 10^−2^	-	-	Solid	[171]
	No added	MgCl_2_	4.76 × 10^−3^	-	1.94	Solid	[172]
Agar	No added	NH_4_SCN	1.03 × 10^−3^	0.25	-	Solid	[173]
	No added	Mg(Tf)_2_	1.0 × 10^−3^	-	-	Solid	[174]
	TiO_2_	LiI	5.12 × 10^−4^	-	-	Solid	[175]

## Data Availability

Not applicable.

## References

[1] Lorca S., Santos F., Fernández Romero A.J. (2020). A review of the use of GPEs in zinc-based batteries. A step closer to wearable electronic gadgets and smart textiles. Polymers.

[2] Jaschin P.W., Gao Y., Li Y., Bo S.H. (2020). A materials perspective on magnesium-ion-based solid-state electrolytes. J. Mater. Chem. A.

[3] Qiu L., Xiang W., Tian W., Xu C.L., Li Y.C., Wu Z.G., Chen T.R., Jia K., Wang D., He F.R. (2019). Polyanion and cation co-doping stabilized Ni-rich Ni–Co–Al material as cathode with enhanced electrochemical performance for Li-ion battery. Nano Energy.

[4] Xu Y.D., Xiang W., Wu Z.G., Xu C.L., Li Y.C., Guo X.D., Lv G.P., Peng X., Zhong B.H. (2018). Improving cycling performance and rate capability of Ni-rich LiNi_0.8_Co_0.1_Mn_0.1_O_2_ cathode materials by Li_4_Ti_5_O_12_ coating. Electrochim. Acta.

[5] Etacheri V., Marom R., Elazari R., Salitra G., Aurbach D. (2011). Challenges in the development of advanced Li-ion batteries: A review. Energy Environ. Sci..

[6] Wang Y., Yi J., Xia Y. (2012). Recent progress in aqueous lithium-ion batteries. Adv. Energy Mater..

[7] Yi J., Wang C., Xia Y. (2013). Comparison of thermal stability between micro- and nano-sized materials for lithium-ion batteries. Electrochem. Commun..

[8] Yi J., Hou M.Y., Bao H.L., Wang C.X., Wang J.Q., Xia Y.Y. (2014). In-situ generation of Li_2_FeSiO_4_/C nanocomposite as cathode material for lithium ion battery. Electrochim. Acta.

[9] Kim H., Hong J., Park K.Y., Kim H., Kim S.W., Kang K. (2014). Aqueous rechargeable Li and Na ion batteries. Chem. Rev..

[10] Wu K., Huang J., Yi J., Liu X., Liu Y., Wang Y., Zhang J., Xia Y. (2020). Recent Advances in Polymer Electrolytes for Zinc Ion Batteries: Mechanisms, Properties, and Perspectives. Adv. Energy Mater..

[11] Xiong P., Zhang L., Chen Y., Peng S., Yu G. (2021). A Chemistry and Microstructure Perspective on Ion-Conducting Membranes for Redox Flow Batteries. Angew. Chemie Int. Ed..

[12] Arévalo-Cid P., Dias P., Mendes A., Azevedo J. (2021). Redox flow batteries: A new frontier on energy storage. Sustain. Energy Fuels.

[13] Zhang H., Sun C. (2021). Cost-effective iron-based aqueous redox flow batteries for large-scale energy storage application: A review. J. Power Sources.

[14] Pankratova G., Bollella P., Pankratov D., Gorton L. (2022). Supercapacitive biofuel cells. Curr. Opin. Biotechnol..

[15] Wang Y., Ruiz Diaz D.F., Chen K.S., Wang Z., Adroher X.C. (2020). Materials, technological status, and fundamentals of PEM fuel cells—A review. Mater. Today.

[16] Wang Y., Seo B., Wang B., Zamel N., Jiao K., Adroher X.C. (2020). Fundamentals, materials, and machine learning of polymer electrolyte membrane fuel cell technology. Energy AI.

[17] Pollock T.M. (2010). Weight loss with magnesium alloys. Science.

[18] Wu X.F., Neumann H. (2012). Zinc-catalyzed organic synthesis: C-C, C-N, C-O bond formation reactions. Adv. Synth. Catal..

[19] Anyadike N. (2015). Lead and Zinc.

[20] USGS (2020). Lithium statistics and information. U.S. Geol. Surv..

[21] Chen M., Ma X., Chen B., Arsenault R., Karlson P., Simon N., Wang Y. (2019). Recycling End-of-Life Electric Vehicle Lithium-Ion Batteries. Joule.

[22] Pan H., Shao Y., Yan P., Cheng Y., Han K.S., Nie Z., Wang C., Yang J., Li X., Bhattacharya P. (2016). Reversible aqueous zinc/manganese oxide energy storage from conversion reactions. Nat. Energy.

[23] Kundu D., Adams B.D., Duffort V., Vajargah S.H., Nazar L.F. (2016). A high-capacity and long-life aqueous rechargeable zinc battery using a metal oxide intercalation cathode. Nat. Energy.

[24] Saha P., Datta M.K., Velikokhatnyi O.I., Manivannan A., Alman D., Kumta P.N. (2014). Rechargeable magnesium battery: Current status and key challenges for the future. Prog. Mater. Sci..

[25] Wang F., Borodin O., Gao T., Fan X., Sun W., Han F., Faraone A., Dura J.A., Xu K., Wang C. (2018). Highly reversible zinc metal anode for aqueous batteries. Nat. Mater..

[26] Deivanayagam R., Ingram B.J., Shahbazian-Yassar R. (2019). Progress in development of electrolytes for magnesium batteries. Energy Storage Mater..

[27] Liu F., Chen Z., Fang G., Wang Z., Cai Y., Tang B., Zhou J., Liang S. (2019). V_2_O_5_ Nanospheres with Mixed Vanadium Valences as High Electrochemically Active Aqueous Zinc-Ion Battery Cathode. Nano-Micro Lett..

[28] Fang G., Zhu C., Chen M., Zhou J., Tang B., Cao X., Zheng X., Pan A., Liang S. (2019). Suppressing Manganese Dissolution in Potassium Manganate with Rich Oxygen Defects Engaged High-Energy-Density and Durable Aqueous Zinc-Ion Battery. Adv. Funct. Mater..

[29] Boaretto N., Meabe L., Martinez-Ibañez M., Armand M., Zhang H. (2020). Review—Polymer Electrolytes for Rechargeable Batteries: From Nanocomposite to Nanohybrid. J. Electrochem. Soc..

[30] Zhao J., Zha J., Zeng Z., Tan C. (2021). Recent advances in wearable self-powered energy systems based on flexible energy storage devices integrated with flexible solar cells. J. Mater. Chem. A.

[31] Volontsevich D., Strimovskyi S., Veretennikov I., Sivykh D., Karpov V. (2022). The Choice of the Electric Energy Storage Device Type for the Hybrid Power Drive of Military Wheeled Vehicles. International Conference Innovation in Engineering.

[32] Luo Y., Wu Y., Li B., Qu J., Feng S.P., Chu P.K. (2021). Optimization and cutting-edge design of fuel-cell hybrid electric vehicles. Int. J. Energy Res..

[33] He W., Zuo S., Xu X., Zeng L., Liu L., Zhao W., Liu J. (2021). Challenges and strategies of zinc anode for aqueous zinc-ion batteries. Mater. Chem. Front..

[34] Ye T., Li L., Zhang Y. (2020). Recent Progress in Solid Electrolytes for Energy Storage Devices. Adv. Funct. Mater..

[35] Zhao C., Liu L., Qi X., Lu Y., Wu F., Zhao J., Yu Y., Hu Y.S., Chen L. (2018). Solid-State Sodium Batteries. Adv. Energy Mater..

[36] Wood K.N., Kazyak E., Chadwick A.F., Chen K.-H., Zhang J.-G., Thornton K., Dasgupta N.P. (2017). Dendrites and Pits: Untangling the Complex Behavior of Li Metal Anodes through Operando Video Microscopy. ECS Meet. Abstr..

[37] Wu F., Yuan Y.X., Cheng X.B., Bai Y., Li Y., Wu C., Zhang Q. (2018). Perspectives for restraining harsh lithium dendrite growth: Towards robust lithium metal anodes. Energy Storage Mater..

[38] Zhang H., Li C., Piszcz M., Coya E., Rojo T., Rodriguez-Martinez L.M., Armand M., Zhou Z. (2017). Single lithium-ion conducting solid polymer electrolytes: Advances and perspectives. Chem. Soc. Rev..

[39] Hallinan D.T., Villaluenga I., Balsara N.P. (2018). Polymer and composite electrolytes. MRS Bull..

[40] Mindemark J., Lacey M.J., Bowden T., Brandell D. (2018). Beyond PEO—Alternative host materials for Li+-conducting solid polymer electrolytes. Prog. Polym. Sci..

[41] Wan J., Xie J., Mackanic D.G., Burke W., Bao Z., Cui Y. (2018). Status, promises, and challenges of nanocomposite solid-state electrolytes for safe and high performance lithium batteries. Mater. Today Nano.

[42] Yu J., Lyu Y.Q., Liu J., Effat M.B., Kwok S.C.T., Wu J., Ciucci F. (2019). Enabling non-flammable Li-metal batteries via electrolyte functionalization and interface engineering. J. Mater. Chem. A.

[43] Weston J.E., Steele B.C.H. (1982). Effects of inert fillers on the mechanical and electrochemical properties of lithium salt-poly(ethylene oxide) polymer electrolytes. Solid State Ionics.

[44] Zhao Q., Stalin S., Zhao C.Z., Archer L.A. (2020). Designing solid-state electrolytes for safe, energy-dense batteries. Nat. Rev. Mater..

[45] Arya A., Sharma A.L. (2019). Electrolyte for energy storage/conversion (Li^+^, Na^+^, Mg^2+^) devices based on PVC and their associated polymer: A comprehensive review. J. Solid State Electrochem..

[46] Park B., Schaefer J.L. (2020). Review—Polymer Electrolytes for Magnesium Batteries: Forging Away from Analogs of Lithium Polymer Electrolytes and Towards the Rechargeable Magnesium Metal Polymer Battery. J. Electrochem. Soc..

[47] Huy V.P.H., So S., Hur J. (2021). Inorganic fillers in composite gel polymer electrolytes for high-performance lithium and non-lithium polymer batteries. Nanomaterials.

[48] Lu K., Jiang T., Hu H., Wu M. (2020). Hydrogel Electrolytes for Quasi-Solid Zinc-Based Batteries. Front. Chem..

[49] Nakajima H., Dijkstra P., Loos K. (2017). The recent developments in biobased polymers toward general and engineering applications: Polymers that are upgraded from biodegradable polymers, analogous to petroleum-derived polymers, and newly developed. Polymers.

[50] Singh R., Polu A.R., Bhattacharya B., Rhee H.W., Varlikli C., Singh P.K. (2016). Perspectives for solid biopolymer electrolytes in dye sensitized solar cell and battery application. Renew. Sustain. Energy Rev..

[51] Rayung M., Aung M.M., Azhar S.C., Abdullah L.C., Su’ait M.S., Ahmad A., Jamil S.N.A.M. (2020). Bio-based polymer electrolytes for electrochemical devices: Insight into the ionic conductivity performance. Materials.

[52] Lizundia E., Kundu D. (2021). Advances in Natural Biopolymer-Based Electrolytes and Separators for Battery Applications. Adv. Funct. Mater..

[53] Austin Suthanthiraraj S., Johnsi M. (2017). Nanocomposite polymer electrolytes. Ionics.

[54] Armand M.B., Bruce P.G., Forsyth M., Scrosati B., Wieczorek W., Bruce D.W., O’Hare D., Walton R.I. (2011). Polymer Electrolytes. Energy Materials.

[55] Abdullah M., Lenggoro W., Okuyama K., Nalwa H.S. (2004). Polymer Electrolyte Nanocomposites. Encyclopedia of Nanoscience and Nanotechnology.

[56] Li Q., Chen J., Fan L., Kong X., Lu Y. (2016). Progress in electrolytes for rechargeable Li-based batteries and beyond. Green Energy Environ..

[57] Johnsi M., Austin Suthanthiraraj S. (2016). Electrochemical and structural properties of a polymer electrolyte system based on the effect of CeO_2_ nanofiller with PVDF-co-HFP for energy storage devices. Ionics.

[58] Abbrent S., Plestil J., Hlavata D., Lindgren J., Tegenfeldt J., Wendsjö Å. (2001). Crystallinity and morphology of PVdF-HFP-based gel electrolytes. Polymer.

[59] Brigandi P.J., Cogen J.M., Pearson R.A. (2014). Electrically conductive multiphase polymer blend carbon-based composites. Polym. Eng. Sci..

[60] Kaur G., Adhikari R., Cass P., Bown M., Gunatillake P. (2015). Electrically conductive polymers and composites for biomedical applications. RSC Adv..

[61] Puguan J.M.C., Chung W.J., Kim H. (2016). Ion-conductive and transparent PVdF-HFP/silane-functionalized ZrO_2_ nanocomposite electrolyte for electrochromic applications. Electrochim. Acta.

[62] Lalia B.S., Guillen E., Arafat H.A., Hashaikeh R. (2014). Nanocrystalline cellulose reinforced PVDF-HFP membranes for membrane distillation application. Desalination.

[63] Prabakaran K., Mohanty S., Nayak S.K. (2015). PEO/PVdF–HFP electrolytes for natural dye sensitized solar cell applications: Effect of modified nano-TiO_2_ on electrochemical and photovoltaic performance. J. Mater. Sci. Mater. Electron..

[64] Shin J., Nho Y.C., seon Hwang I., Fei G., Kim A.R., Nahm K.S. (2010). Irradiated PVdF-HFP-tin oxide composite membranes for the applications of direct methanol fuel cells. J. Memb. Sci..

[65] Xie L., Huang X., Yang K., Li S., Jiang P. (2014). “Grafting to” route to PVDF-HFP-GMA/BaTiO_3_ nanocomposites with high dielectric constant and high thermal conductivity for energy storage and thermal management applications. J. Mater. Chem. A.

[66] Zhu L., Wang Q. (2012). Novel ferroelectric polymers for high energy density and low loss dielectrics. Macromolecules.

[67] Xiong M., Tang H., Wang Y., Lin Y., Sun M., Yin Z., Pan M. (2013). Expanded polytetrafluoroethylene reinforced polyvinylidenefluoride- hexafluoropropylene separator with high thermal stability for lithium-ion batteries. J. Power Sources.

[68] Ataollahi N., Ahmad A., Hamzah H., Rahman M.Y.A., Mohamed N.S. (2012). Preparation and Characterization of PVDF-HFP/MG49 Based Polymer Blend Electrolyte. Int. J. Electrochem. Sci.

[69] Ma T., Cui Z., Wu Y., Qin S., Wang H., Yan F., Han N., Li J. (2013). Preparation of PVDF based blend microporous membranes for lithium ion batteries by thermally induced phase separation: I. Effect of PMMA on the membrane formation process and the properties. J. Memb. Sci..

[70] Yang Q., Deng N., Chen J., Cheng B., Kang W. (2020). The recent research progress and prospect of gel polymer electrolytes in lithium-sulfur batteries. Chem. Eng. J..

[71] Zhou H. (2013). New energy storage devices for post lithium-ion batteries. Energy Environ. Sci..

[72] Maheshwaran C., Mishra K., Kanchan D.K., Kumar D. (2020). Mg^2+^ conducting polymer gel electrolytes: Physical and electrochemical investigations. Ionics.

[73] Ponmani S., Prabhu M.R. (2018). Development and study of solid polymer electrolytes based on PVdF-HFP/PVAc: Mg (ClO_4_)_2_ for Mg ion batteries. J. Mater. Sci. Mater. Electron..

[74] Oh J.S., Ko J.M., Kim D.W. (2004). Preparation and characterization of gel polymer electrolytes for solid state magnesium batteries. Electrochim. Acta.

[75] Pandey G.P., Agrawal R.C., Hashmi S.A. (2011). Performance studies on composite gel polymer electrolytes for rechargeable magnesium battery application. J. Phys. Chem. Solids.

[76] Pandey G.P., Agrawal R.C., Hashmi S.A. (2011). Magnesium ion-conducting gel polymer electrolytes dispersed with fumed silica for rechargeable magnesium battery application. J. Solid State Electrochem..

[77] Sharma J., Hashmi S. (2019). Magnesium ion-conducting gel polymer electrolyte nanocomposites: Effect of active and passive nanofillers. Polym. Compos..

[78] Patel S., Kumar R. (2021). Effect of Al_2_O_3_ on electrical properties of polymer electrolyte for electrochemical device application. Mater. Today Proc..

[79] Pandey G.P., Agrawal R.C., Hashmi S.A. (2009). Magnesium ion-conducting gel polymer electrolytes dispersed with nanosized magnesium oxide. J. Power Sources.

[80] Patel S., Kumar R. (2019). Synthesis and characterization of magnesium ion conductivity in PVDF based nanocomposite polymer electrolytes disperse with MgO. J. Alloys Compd..

[81] Pandey G.P., Agrawal R.C., Hashmi S.A. (2010). Electrical and electrochemical properties of magnesium ion conducting composite gel polymer electrolytes. J. Phys. D. Appl. Phys..

[82] Nidhi Sandhya P., Kumar R. (2020). PVDF-HFP based nanocomposite polymer electrolytes for energy storage devices dispersed with various nano-fillers. AIP Conf. Proc..

[83] Patel S., Kumar R. (2021). Effect of nanoparticles on electrical properties of PVDF-based Mg^2+^ ion conducting polymer electrolytes. Bull. Mater. Sci..

[84] Jayanthi S., Kalapriya K. (2021). Structural, transport, morphological, and thermal studies of nano barium titanate–incorporated magnesium ion conducting solid polymer electrolytes. Polym. Polym. Compos..

[85] Patel S., Kumar R. (2021). Effect of dispersion of ceramic filler on thermal, structural and transport properties of polymer electrolyte for electrochemical applications. AIP Conf. Proc..

[86] Kumar B. (2004). From colloidal to composite electrolytes: Properties, peculiarities, and possibilities. J. Power Sources.

[87] Polu A.R., Kumar R., Joshi G.M. (2014). Effect of zinc salt on transport, structural, and thermal properties of PEG-based polymer electrolytes for battery application. Ionics.

[88] Tafur J.P., Fernández Romero A.J. (2014). Electrical and spectroscopic characterization of PVdF-HFP and TFSI-ionic liquids-based gel polymer electrolyte membranes. Influence of ZnTf_2_ salt. J. Memb. Sci..

[89] Liu J., Khanam Z., Muchakayala R., Song S. (2020). Fabrication and characterization of Zn-ion-conducting solid polymer electrolyte films based on PVdF-HFP/Zn(Tf)_2_ complex system. J. Mater. Sci. Mater. Electron..

[90] Liu J., Ahmed S., Khanam Z., Wang T., Song S. (2020). Ionic liquid-incorporated zn-ion conducting polymer electrolyte membranes. Polymers.

[91] Johnsi M., Suthanthiraraj S.A. (2015). Preparation, zinc ion transport properties, and battery application based on poly(vinilydene fluoride-*co*-hexa fluoro propylene) polymer electrolyte system containing titanium dioxide nanofiller. High Perform. Polym..

[92] Johnsi M., Suthanthiraraj S.A. (2016). Compositional effect of ZrO_2_ nanofillers on a PVDF-*co*-HFP based polymer electrolyte system for solid state zinc batteries. Chin. J. Polym. Sci..

[93] Hashmi S.A. (2012). Enhanced zinc ion transport in gel polymer electrolyte: Effect of nano-sized ZnO dispersion. J. Solid State Electrochem..

[94] Muda N., Ibrahim S., Kamarulzaman N., Mohamed N.S. (2011). PVDF-HFP-NH_4_CF_3_SO_3_-SiO_2_ nanocomposite polymer electrolytes for protonic electrochemical cell. Key Eng. Mater..

[95] Tafur J.P., Abad J., Román E., Fernández Romero A.J. (2015). Charge storage mechanism of MnO_2_ cathodes in Zn/MnO_2_ batteries using ionic liquid-based gel polymer electrolytes. Electrochem. Commun..

[96] Jaipal Reddy M., Chu P.P. (2002). Ion pair formation and its effect in PEO:Mg solid polymer electrolyte system. J. Power Sources.

[97] Agrawal R.C., Pandey G.P. (2008). Solid polymer electrolytes: Materials designing and all-solid-state battery applications: An overview. J. Phys. D. Appl. Phys..

[98] Prodduturi S., Manek R.V., Kolling W.M., Stodghill S.P., Repka M.A. (2005). Solid-State Stability and Characterization of Hot-Melt Extruded Poly(ethylene oxide) Films. J. Pharm. Sci..

[99] Chawla P., Trivedi S., Pandey K., Tripathi M. (2018). Dielectric Studies of [PEO: CH3COOLi]: Graphite System Synthesized by Hot Press and Solution Cast Technique. Proc. Natl. Acad. Sci. India Sect. A Phys. Sci..

[100] Lascaud S., Perrier M., Vallee A., Besner S., Prud’homme J., Armand M. (1994). Phase Diagrams and Conductivity Behavior of Poly(ethylene oxide)-Molten Salt Rubbery Electrolytes. Macromolecules.

[101] Agrawal R.C., Sahu D.K., Mahipal Y.K., Ashrafi R. (2013). Investigations on ion transport properties of hot-press cast magnesium ion conducting Nano-Composite Polymer Electrolyte (NCPE) films: Effect of filler particle dispersal on room temperature conductivity. Mater. Chem. Phys..

[102] Feng J., Wang L., Chen Y., Wang P., Zhang H., He X. (2021). PEO based polymer-ceramic hybrid solid electrolytes: A review. Nano Converg..

[103] Kumar Y., Hashmi S.A., Pandey G.P. (2011). Ionic liquid mediated magnesium ion conduction in poly(ethylene oxide) based polymer electrolyte. Electrochim. Acta.

[104] Agrawal R.C., Sahu D.K., Mahipal Y.K., Ashrafi R. (2013). Ion transport property of hot-press cast Mg^2+^-ion conducting nano-composite polymer electrolyte membranes: Study of effect of active/passive filler particle dispersal on conductivity. Indian J. Pure Appl. Phys..

[105] Zaky M.M., Eyssa H.M., Sadek R.F. (2019). Improvement of the magnesium battery electrolyte properties through gamma irradiation of nano polymer electrolytes doped with magnesium oxide nanoparticles. J. Vinyl Addit. Technol..

[106] Sundar M., Selladurai S. (2006). Effect of fillers on magnesium-poly(ethylene oxide) solid polymer electrolyte. Ionics.

[107] Koduru H.K., Marinov Y.G., Kaleemulla S., Rafailov P.M., Hadjichristov G.B., Scaramuzza N. (2021). Fabrication and characterization of magnesium—ion-conducting flexible polymer electrolyte membranes based on a nanocomposite of poly(ethylene oxide) and potato starch nanocrystals. J. Solid State Electrochem..

[108] Shao Y., Rajput N.N., Hu J., Hu M., Liu T., Wei Z., Gu M., Deng X., Xu S., Han K.S. (2015). Nanocomposite polymer electrolyte for rechargeable magnesium batteries. Nano Energy.

[109] Carrilho-Plancha M.J., Rangel C.M., Correia De Sequeira C.A. (2012). Electrochemical characterisation of a Zn/(PEO)_4_ZnCl_2_/Nb_2_O_5_ solid-state cell. J. Solid State Electrochem..

[110] Nancy A.C., Suthanthiraraj S.A. (2017). Effect of Al2O3 nanofiller on the electrical, thermal and structural properties of PEO:PPG based nanocomposite polymer electrolyte. Ionics.

[111] Karan S., Agrawal R.C. (2019). Ion Transport and Materials Characterization Studies on Hot-Press Cast Zn^2+^ Conducting Nano-Composite Polymer Electrolyte (NCPE) Films: [90 PEO: 10Zn (CF_3_SO_3_)_2_] + xAl_2_O_3_. J. Ravishankar Univ..

[112] Turković A., Pavlović M., Dubček P., Lučić-Lavčević M., Etlinger B., Bernstorff S. (2007). SAXS/DSC Study of Polymer Electrolyte for Zn Rechargeable Nanostructured Galvanic Cells. J. Electrochem. Soc..

[113] Turković A., Dubček P., Pavlović M., Bernstorff S. (2009). SAXS/DSC/WAXD Study of γ-irradiated Polymer Electrolyte for Zn Rechargeable Nanostructured Galvanic Cells. ECS Trans..

[114] Agrawal R.C., Hashmi S.A., Pandey G.P. (2007). Electrochemical cell performance studies on all-solid-state battery using nano-composite polymer electrolyte membrane. Ionics (Kiel).

[115] Karan S., Sahu M., Sahu T.B., Mahipal Y.K., Sahu D.K., Agrawal R.C. (2017). Investigations on materials and ion transport properties of Zn^2+^ conducting nano-composite polymer electrolytes (NCPEs): [(90 PEO: 10Zn(CF_3_SO_3_)_2_)+ xZnO]. Mater. Today Commun..

[116] Agrawal S.L., Singh M., Dwivedi M.M., Pandey K. (2011). Investigation on ion conduction behaviour in Zn-ferrite based polymer nanocomposite electrolyte. Fibers Polym..

[117] Wang M., Emre A., Tung S., Gerber A., Wang D., Huang Y., Cecen V., Kotov N.A. (2019). Biomimetic Solid-State Zn2+ Electrolyte for Corrugated Structural Batteries. ACS Nano.

[118] Liu J., Guan C., Zhou C., Fan Z., Ke Q., Zhang G., Liu C., Wang J. (2016). A Flexible Quasi-Solid-State Nickel–Zinc Battery with High Energy and Power Densities Based on 3D Electrode Design. Adv. Mater..

[119] Li H., Han C., Huang Y., Huang Y., Zhu M., Pei Z., Xue Q., Wang Z., Liu Z., Tang Z. (2018). An extremely safe and wearable solid-state zinc ion battery based on a hierarchical structured polymer electrolyte. Energy Environ. Sci..

[120] Fu J., Zhang J., Song X., Zarrin H., Tian X., Qiao J., Rasen L., Li K., Chen Z. (2016). A flexible solid-state electrolyte for wide-scale integration of rechargeable zinc–air batteries. Energy Environ. Sci..

[121] Li Q., Sun H.Y., Takeda Y., Imanishi N., Yang J., Yamamoto O. (2001). Interface properties between a lithium metal electrode and a poly(ethylene oxide) based composite polymer electrolyte. J. Power Sources.

[122] Rao C.V.S., Ravi M., Raja V., Bhargav P.B., Sharma A.K., Rao V.V.R.N. (2012). Preparation and characterization of PVP-based polymer electrolytes for solid-state battery applications. Iran. Polym. J..

[123] CROW Polymer Database. http://polymerdatabase.com/index.html.

[124] Sarojini S., Anjalai C. (2016). AC Impedance Studies on Magnesium Ion Conducting Polymer Electrolyte System with Ethylene Carbonate as Plasticizer and MgO as Nanofiller. Chem. Sci. Trans..

[125] Zain N.F., Zainal N., Mohamed N.S. (2018). The effects of MgO nanofiller to the physicochemical and ionic liquid retention properties of PEMA-MgTf2-EMITFSI nanocomposite polymer electrolytes. Polym. Compos..

[126] Shahenoor Basha S.K., Sunita Sundari G., Vijay Kumar K., Rao M.C. (2017). Optical and dielectric properties of PVP based composite polymer electrolyte films. Polym. Sci. Ser. A.

[127] Mishra K., Hashmi S.A., Rai D.K. (2013). Nanocomposite blend gel polymer electrolyte for proton battery application. J. Solid State Electrochem..

[128] Candhadai Murali S.P., Samuel A.S. (2019). Zinc ion conducting blended polymer electrolytes based on room temperature ionic liquid and ceramic filler. J. Appl. Polym. Sci..

[129] Sai Prasanna C.M., Austin Suthanthiraraj S. (2019). Investigations of Zinc Ion Dissociation in Gel Polymer Electrolytes Based on Poly(vinyl chloride) and Poly(ethyl methacrylate) Blend on the Addition of Two Different Ceramic Nanofillers. J. Inorg. Organomet. Polym. Mater..

[130] Sai Prasanna C.M., Austin Suthanthiraraj S. (2019). PVC/PEMA-based blended nanocomposite gel polymer electrolytes plasticized with room temperature ionic liquid and dispersed with nano-ZrO_2_ for zinc ion batteries. Polym. Compos..

[131] Sai Prasanna C.M., Austin Suthanthiraraj S. (2020). Improved zinc ion transportation in gel polymer electrolyte upon the addition of nano-sized SnO_2_. Polym. Polym. Compos..

[132] Sownthari K., Suthanthiraraj S.A. (2015). Preparation and properties of biodegradable polymer-layered silicate nanocomposite electrolytes for zinc based batteries. Electrochim. Acta.

[133] Sownthari K., Suthanthiraraj S.A. (2014). Structural and AC impedance studies on nanocomposite polymer electrolytes based on poly(ε-caprolactone). J. Appl. Polym. Sci..

[134] Fan X., Liu J., Song Z., Han X., Deng Y., Zhong C., Hu W. (2019). Porous nanocomposite gel polymer electrolyte with high ionic conductivity and superior electrolyte retention capability for long-cycle-life flexible zinc–air batteries. Nano Energy.

[135] Abdullah O.G., Salman Y.A.K., Tahir D.A., Jamal G.M., Ahmed H.T., Mohamad A.H., Azawy A.K., Abdullah C., Salman O.G., Tahir Y.A.K. (2021). Effect of ZnO nanoparticle content on the structural and ionic transport parameters of polyvinyl alcohol based proton-conducting polymer electrolyte membranes. Membranes.

[136] Chen H.W., Lin T.P., Chang F.C. (2002). Ionic conductivity enhancement of the plasticized PMMA/LiCIO4 polymer nanocomposite electrolyte containing clay. Polymer (Guildf).

[137] Jäger M., Wilke A. (2003). Comprehensive biocompatibility testing of a new PMMA-HA bone cement versus conventional PMMA cement in vitro. J. Biomater. Sci. Polym. Ed..

[138] Kikuchi Y., Hirao M., Ookubo T., Sasaki A. (2014). Design of recycling system for poly(methyl methacrylate) (PMMA). Part 1: Recycling scenario analysis. Int. J. Life Cycle Assess..

[139] Su’Ait M.S., Ahmad A., Hamzah H., Rahman M.Y.A. (2009). Preparation and characterization of PMMA-MG49-LiClO_4_ solid polymeric electrolyte. J. Phys. D. Appl. Phys..

[140] Ahmad S., Ahmad S., Agnihotry S.A. (2005). Nanocomposite electrolytes with fumed silica in poly(methyl methacrylate): Thermal, rheological and conductivity studies. J. Power Sources.

[141] Han H.S., Kang H.R., Kim S.W., Kim H.T. (2002). Phase-separated polymer electrolyte based on poly(vinyl chloride)/poly(ethyl methacrylate) blend. J. Power Sources.

[142] Reiter J., Krejza O., Sedlaříková M. (2009). Electrochromic devices employing methacrylate-based polymer electrolytes. Sol. Energy Mater. Sol. Cells.

[143] Turner D.T., Schwartz A. (1985). The glass transition temperature of poly(N-vinyl pyrrolidone) by differential scanning calorimetry. Polymer.

[144] Ramaswamy M., Malayandi T., Subramanian S., Srinivasalu J., Rangaswamy M. (2017). Magnesium ion conducting polyvinyl alcohol–polyvinyl pyrrolidone-based blend polymer electrolyte. Ionics.

[145] Majhi P.R., Moulik S.P., Burke S.E., Rodgers M., Palepu R. (2001). Physicochemical investigations on the interaction of surfactants and salts with polyvinylpyrrolidone in aqueous medium. J. Colloid Interface Sci..

[146] Rajendran S., Sivakumar M., Subadevi R. (2004). Investigations on the effect of various plasticizers in PVA-PMMA solid polymer blend electrolytes. Mater. Lett..

[147] Dubal D.P., Chodankar N.R., Kim D.H., Gomez-Romero P. (2018). Towards flexible solid-state supercapacitors for smart and wearable electronics. Chem. Soc. Rev..

[148] Wang Z., Meng X., Wu Z., Mitra S. (2017). Development of flexible zinc–air battery with nanocomposite electrodes and a novel separator. J. Energy Chem..

[149] Aziz S.B. (2013). Li+ ion conduction mechanism in poly (ε-caprolactone)-based polymer electrolyte. Iran. Polym. J..

[150] Flieger M., Kantorová M., Prell A., Řezanka T., Votruba J. (2003). Biodegradable plastics from renewable sources. Folia Microbiol..

[151] Ray S.S., Bousmina M. (2005). Biodegradable polymers and their layered silicate nanocomposites: In greening the 21st century materials world. Prog. Mater. Sci..

[152] Salleh N.S., Aziz S.B., Aspanut Z., Kadir M.F.Z. (2016). Electrical impedance and conduction mechanism analysis of biopolymer electrolytes based on methyl cellulose doped with ammonium iodide. Ionics.

[153] Huang X., Wang D., Yuan Z., Xie W., Wu Y., Li R., Zhao Y., Luo D., Cen L., Chen B. (2018). A Fully Biodegradable Battery for Self-Powered Transient Implants. Small.

[154] Majdecka D., Drami ska S., Stolarczyk K., Kizling M., Krysiski P., Golimowski J., Bilewicz R. (2014). Sandwich Biobattery with Enzymatic Cathode and Zinc Anode for Powering Sensors. ECS Trans..

[155] Huang X. (2018). Materials and applications of bioresorbable electronics. J. Semicond..

[156] Poosapati A., Jang E., Madan D., Jang N., Hu L., Lan Y. (2019). Cellulose hydrogel as a flexible gel electrolyte layer. MRS Commun..

[157] Johari N.A., Kudin T.I.T., Ali A.M.M., Winie T., Yahya M.Z.A. (2009). Studies on cellulose acetate-based gel polymer electrolytes for proton batteries. Mater. Res. Innov..

[158] Johari N.A., Kudin T.I.T., Ali A.M.M., Yahya M.Z.A. (2011). Effects of TiO_2_ on conductivity performance of cellulose acetate based polymer gel electrolytes for proton batteries. Mater. Res. Innov..

[159] Muhammad F.H., Subban R.H.Y., Winie T. (2009). Electrical studies on hexanoyl chitosan-based nanocomposite polymer electrolytes. AIP Conf. Proc..

[160] Muhammad F.H., Subban R.H.Y., Winie T. (2014). Structural and electrical characterization of hexanoyl chitosan- LiClO_4_-TiO_2_-DMC polymer electrolytes. Key Eng. Mater..

[161] Rosli N.H.A., Muhammad F.H., Chan C.H., Winie T. (2014). Effect of filler type on the electrical properties of hexanoyl chitosan- based polymer electrolytes. Adv. Mater. Res..

[162] Aziz N.A., Majid S.R., Yahya R., Arof A.K. (2011). Conductivity, structure, and thermal properties of chitosan-based polymer electrolytes with nanofillers. Polym. Adv. Technol..

[163] Navaratnam S., Ramesh K., Basirun W.J. (2011). Investigation of ion conducting behaviour of composite chitosan based polymer electrolytes. Mater. Res. Innov..

[164] Sudaryanto, Yulianti E., Patimatuzzohrah (2016). Structure and properties of solid polymer electrolyte based on chitosan and ZrO_2_ nanoparticle for lithium ion battery. AIP Conf. Proc..

[165] Shukur M.F., Ithnin R., Kadir M.F.Z. (2016). Ionic conductivity and dielectric properties of potato starch-magnesium acetate biopolymer electrolytes: The effect of glycerol and 1-butyl-3-methylimidazolium chloride. Ionics.

[166] Khanmirzaei M.H., Ramesh S. (2014). Nanocomposite polymer electrolyte based on rice starch/ionic liquid/TiO_2_ nanoparticles for solar cell application. Meas. J. Int. Meas. Confed..

[167] Teoh K.H., Ramesh S., Arof A.K. (2012). Investigation on the effect of nanosilica towards corn starch-lithium perchlorate-based polymer electrolytes. J. Solid State Electrochem..

[168] Teoh K.H., Lim C.S., Liew C.W., Ramesh S., Ramesh S. (2015). Electric double-layer capacitors with corn starch-based biopolymer electrolytes incorporating silica as filler. Ionics.

[169] Shukur M.F., Kadir M.F.Z. (2015). Hydrogen ion conducting starch-chitosan blend based electrolyte for application in electrochemical devices. Electrochim. Acta.

[170] Masri M.N., Nazeri M.F.M., Mohamad A.A. (2010). Sago Gel Polymer Electrolyte for Zinc-Air Battery. Adv. Sci. Technol..

[171] Huang Y., Liu J., Zhang J., Jin S., Jiang Y., Zhang S., Li Z., Zhi C., Du G., Zhou H. (2019). Flexible quasi-solid-state zinc ion batteries enabled by highly conductive carrageenan bio-polymer electrolyte. RSC Adv..

[172] Sangeetha P., Selvakumari T.M., Selvasekarapandian S., Srikumar S.R., Manjuladevi R., Mahalakshmi M. (2020). Preparation and characterization of biopolymer K-carrageenan with MgCl_2_ and its application to electrochemical devices. Ionics.

[173] Selvalakshmi S., Vijaya N., Selvasekarapandian S., Premalatha M. (2017). Biopolymer agar-agar doped with NH4SCN as solid polymer electrolyte for electrochemical cell application. J. Appl. Polym. Sci..

[174] Alves R.D., Rodrigues L.C., Andrade J.R., Pawlicka A., Pereira L., Martins R., Fortunato E., Silva M.M. (2013). Study and characterization of a novel polymer electrolyte based on agar doped with magnesium triflate. Mol. Cryst. Liq. Cryst..

[175] Wang W., Guo X., Yang Y. (2011). Lithium iodide effect on the electrochemical behavior of agarose based polymer electrolyte for dye-sensitized solar cell. Electrochim. Acta.

[176] Sudhakar Y.N., Selvakumar M.D., Krishna B. (2018). Biopolymer Electrolytes Fundamentals and Applications in Energy Storage.

[177] Wang W., Zhang X., Teng A., Liu A. (2017). Mechanical reinforcement of gelatin hydrogel with nanofiber cellulose as a function of percolation concentration. Int. J. Biol. Macromol..

[178] Zhang J., Fu J., Song X., Jiang G., Zarrin H., Xu P., Li K., Yu A., Chen Z. (2016). Laminated Cross-Linked Nanocellulose/Graphene Oxide Electrolyte for Flexible Rechargeable Zinc–Air Batteries. Adv. Energy Mater..

[179] Yahya M.Z.A., Arof A.K. (2003). Effect of oleic acid plasticizer on chitosan-lithium acetate solid polymer electrolytes. Eur. Polym. J..

[180] Yang R., Li H., Huang M., Yang H., Li A. (2016). A review on chitosan-based flocculants and their applications in water treatment. Water Res..

[181] Mohamed N.S., Subban R.H.Y., Arof A.K. (1995). Polymer batteries fabricated from lithium complexed acetylated chitosan. J. Power Sources.

[182] Winie T., Jamal A., Hanif N.S.M., Shahril N.S.M. (2014). Hexanoyl chitosan-polystyrene blend based composite polymer electrolyte with surface treated TiO2 fillers. Key Eng. Mater..

[183] Winie T., Hanif N.S.M., Chan C.H., Arof A.K. (2014). Effect of the surface treatment of the TiO_2_ fillers on the properties of hexanoyl chitosan/polystyrene blend-based composite polymer electrolytes. Ionics.

[184] Winie T., Mohd Shahril N.S. (2015). Conductivity enhancement by controlled percolation of inorganic salt in multiphase hexanoyl chitosan/polystyrene polymer blends. Front. Mater. Sci..

[185] Aziz S.B., Rasheed M.A., Abidin Z.H.Z. (2017). Optical and Electrical Characteristics of Silver Ion Conducting Nanocomposite Solid Polymer Electrolytes Based on Chitosan. J. Electron. Mater..

[186] Aziz S.B. (2016). Role of dielectric constant on ion transport: Reformulated Arrhenius equation. Adv. Mater. Sci. Eng..

[187] Aziz S.B., Abidin Z.H.Z. (2015). Ion-transport study in nanocomposite solid polymer electrolytes based on chitosan: Electrical and dielectric analysis. J. Appl. Polym. Sci..

[188] Muhammad F.H., Azmar A., Winie T. (2015). Transport properties of hexanoyl chitosan-LiClO_4_-TiO_2_ composite polymer electrolyte. AIP Conf. Proc..

[189] Rosli N.H.A., Muhammad F.H., Subban R.H.Y., Winie T. (2011). Structural and electrical studies of hexanoyl chitosan based electrolyte system. Mater. Res. Innov..

[190] Winie T., Han C.C., Subban R.H.Y. (2011). Ac conductivity and dielectric properties of hexanoyl chitosan-LiClO_4_-TiO_2_ composite polymer electrolytes. Adv. Mater. Res..

[191] Winie T., Hanif N.S.M., Rosli N.H.A., Subban R.H.Y. (2013). Ac Conductivity Study of Hexanoyl Chitosan-LiCF_3_SO_3_-EC-Al_2_O_3_ Nanocomposite Polymer Electrolytes. Adv. Mater. Res..

[192] Wang J., Song S., Gao S., Muchakayala R., Liu R., Ma Q. (2017). Mg-ion conducting gel polymer electrolyte membranes containing biodegradable chitosan: Preparation, structural, electrical and electrochemical properties. Polym. Test..

[193] Dannoun E.M.A., Aziz S.B., Brza M.A., Nofal M.M., Asnawi A.S.F.M., Yusof Y.M., Al-Zangana S., Hamsan M.H., Kadir M.F.Z., Woo H.J. (2020). The study of plasticized solid polymer blend electrolytes based on natural polymers and their application for energy storage EDLC devices. Polymers.

[194] Aziz S.B., Dannoun E.M.A., Hamsan M.H., Abdulwahid R.T., Mishra K., Nofal M.M., Kadir M.F.Z., Appetecchi B., Kim D. (2021). Improving EDLC Device Performance Constructed from Plasticized Magnesium Ion Conducting Chitosan Based Polymer Electrolytes via Metal Complex Dispersion. Membranes.

[195] Hamsan M.H., Aziz S.B., Nofal M.M., Brza M.A., Abdulwahid R.T., Hadi J.M., Karim W.O., Kadir M.F.Z. (2020). Characteristics of EDLC device fabricated from plasticized chitosan:MgCl_2_ based polymer electrolyte. J. Mater. Res. Technol..

[196] Pawlicka A., Sabadini A.C., Raphael E., Dragunski D.C. (2008). Ionic conductivity thermogravimetry measurements of starch-based polymeric electrolytes. Mol. Cryst. Liq. Cryst..

[197] Khiar A.S.A., Arof A.K. (2010). Conductivity studies of starch-based polymer electrolytes. Ionics.

[198] Sen A., Bhattacharya M. (2000). Residual stresses and density gradient in injection molded starch/synthetic polymer blends. Polymer.

[199] Wang J., Liang Y., Zhang Z., Ye C., Chen Y., Wei P., Wang Y., Xia Y. (2021). Thermoplastic starch plasticized by polymeric ionic liquid. Eur. Polym. J..

[200] Zahid A.R.M., Masri M.N., Hussin M.H., Bakar M.B.A. (2018). The preliminary study on cassava (*Manihot esculenta*) as gel polymer electrolyte for zinc-air battery. AIP Conf. Proc..

[201] Mobarak N.N., Jumaah F.N., Ghani M.A., Abdullah M.P., Ahmad A. (2015). Carboxymethyl Carrageenan Based Biopolymer Electrolytes. Electrochim. Acta.

[202] Moniha V., Alagar M., Selvasekarapandian S., Sundaresan B., Boopathi G. (2018). Conductive bio-polymer electrolyte iota-carrageenan with ammonium nitrate for application in electrochemical devices. J. Non. Cryst. Solids.

[203] De Ruiter G.A., Rudolph B. (1997). Carrageenan biotechnology. Trends Food Sci. Technol..

[204] Yao Z., Wu H., Zhang S., Du Y. (2014). Enzymatic preparation of κ-carrageenan oligosaccharides and their anti-angiogenic activity. Carbohydr. Polym..

[205] Pacheco-Quito E.M., Ruiz-Caro R., Veiga M.D. (2020). Carrageenan: Drug Delivery Systems and Other Biomedical Applications. Mar. Drugs.

[206] Sabbagh F., Kiarostami K., Khatir N.M., Rezania S., Muhamad I.I., Hosseini F. (2021). Effect of zinc content on structural, functional, morphological, and thermal properties of kappa-carrageenan/NaCMC nanocomposites. Polym. Test..

[207] Chan S., Paolo J., Bantang O., Bantang J.P., Camacho D. (2015). Influence of Nanomaterial Fillers in Biopolymer Electrolyte System for Squaraine-based Dye-Sensitized Solar Cells. Int. J. Electrochem. Sci.

[208] Armisén R., Galatas F. (2009). Agar. Handbook of Hydrocolloids: Second Edition.

[209] Kato S., Yamagishi A., Daimon S., Kawasaki K., Tamaki H., Kitagawa W., Abe A., Tanaka M., Sone T., Asano K. (2018). Isolation of previously uncultured slowgrowing bacteria by using a simple modification in the preparation of agar media. Appl. Environ. Microbiol..

[210] Selvalakshmi S., Mathavan T., Selvasekarapandian S., Premalatha M. (2018). Effect of ethylene carbonate plasticizer on agar-agar: NH4Br-based solid polymer electrolytes. Ionics.

[211] An L., Zhao T.S., Zeng L. (2013). Agar chemical hydrogel electrode binder for fuel-electrolyte-fed fuel cells. Appl. Energy.

[212] Yang Y., Hu H., Zhou C.H., Xu S., Sebo B., Zhao X.Z. (2011). Novel agarose polymer electrolyte for quasi-solid state dye-sensitized solar cell. J. Power Sources.

